# Clade 2.3.4.4b H5N8 Subtype Avian Influenza Viruses Were Identified from the Common Crane Wintering in Yunnan Province, China

**DOI:** 10.3390/v15010038

**Published:** 2022-12-22

**Authors:** Qinhong Yang, Xiaoyan Xue, Zhenxing Zhang, Ming J. Wu, Jia Ji, Wei Wang, Hongbin Yin, Suhua Li, Hongyang Dai, Bofang Duan, Qiang Liu, Jianling Song

**Affiliations:** 1 College of Life Sciences, Southwest Forestry University, 300 Bailong Road, Kunming 650024, China; 2 Yunnan Tropical and Subtropical Animal Virus Diseases Laboratory, Yunnan Academy of Animal Husbandry and Veterinary, 6 Qinglongshan, Kunming 650224, China; 3 School of Science, Western Sydney University, Locked Bag 1797, Penrith, NSW 2751, Australia; 4 Animal Disease Inspection and Supervision Institution of Yunnan Province, 118 Gulou Road, Kunming 650051, China; 5 The Management Bureau of Huize Black—Necked Crane National Nature Reserve, 744 Tongbao Road, Qujing 654200, China; 6 Yunnan Center for Animal Disease Control and Prevention, 95 Jinhei Road, Kunming 650034, China

**Keywords:** avian influenza virus, H5N8, wild bird, genetic analysis

## Abstract

The seasonal migration of wild aquatic birds plays a critical role in the maintenance, transmission, and incursion of the avian influenza virus (AIV). AIV surveillance was performed during 2020–2021 in two national nature reserves with abundant wild bird resources in Yunnan, China. Four H5N8 AIVs isolates from the common crane were identified by next-generation sequencing. Phylogenetic analysis demonstrated that all eight gene segments of these H5N8 AIVs belonged to clade 2.3.4.4b high-pathogenic AIV (HPAIV) and shared high nucleotide sequence similarity with the strains isolated in Hubei, China, and Siberia, Russia, in 2020–2021. The H5N8 HPAIVs from common cranes were characterized by both human and avian dual-receptor specificity in the hemagglutinin (HA) protein. Moreover, possessing the substitutions contributes to overcoming transmission barriers of mammalian hosts in polymerase basic 2 (PB2), polymerase basic protein 1 (PB1), and polymerase acid (PA), and exhibiting the long stalk in the neck region of the neuraminidase (NA) protein contributes to adaptation in wild birds. Monitoring AIVs in migratory birds, at stopover sites and in their primary habitats, i.e., breeding or wintering grounds, could provide insight into potential zoonosis caused by AIVs.

## 1. Introduction

Wild birds, particularly aquatic birds belonging to the orders Anseriformes and Charadriiformes, are considered to serve as the main natural reservoir of the avian influenza virus (AIV) and play a role in viral maintenance and transmission. The natural hosts are usually asymptomatic or mildly symptomatic after being affected by low pathogenic AIVs (LPAIVs). However, LPAIVs can replicate in the intestinal epithelial cells of wild birds and persistently shed virus through cloaca [1]. The feces of wild aquatic birds containing high viral loads can contaminate aquatic habitats, facilitating fecal–oral AIV transmission [1]. It was accepted that high-pathogenic AIVs (HPAIVs) are not normally present in wild bird hosts, until novel H5N1 HPAIV (A/goose/Guangdong/1/1996, or Gs/GD) was identified in a domestic goose in Guangdong Province, China, in 1996, which subsequently caused significant mortality in wild birds in 2006 [2,3].

H5N1 HPAIV, which bears a hemagglutinin (*HA*) segment of H5, acquired various neuraminidase (*NA*) genes, including N2, N5, N6, and N8, from the prevailing LPAIVs in wild aquatic birds from different regions by gene reassortment during viral dissemination and evolved into a distinct monophyletic lineage [4,5]. The H5N1, H5N2, H5N5, H5N6, and H5N8 subtypes (known as H5Nx) were classified in clade 2.3.4.4 as the unified classification by the WHO/OIE/FAO H5 Evolution Working Group [6,7]. According to the spatiotemporal antigenic and genetic characteristics of the viral HA protein, the circulating clade 2.3.4.4 H5Nx HPAIVs evolved into eight groups, namely, clade 2.3.4.4a-h [8].

Clade 2.3.4.4b H5N8 HPAIVs spread extremely rapidly and, to date, have caused three worldwide outbreaks, resulting in high mortality in waterfowl and domestic birds [9,10,11,12,13]. The H5N8 subtype was initially identified from apparently healthy ducks (*Annas platyrhynchos*) in Jiangsu Province, East China, in 2010, as represented by A/duck/Jiangsu/k1203/2010 [14], whereas there are no records of its circulation in China or the regions outside China before 2014. Two distinct genetic clades, i.e., clade 2.3.4.4a H5N8 HPAIV (A/broiler duck/Korea/Buan 2/2014, referred to as the Buan-like group) and clade 2.3.4.4b H5N8 HPAIV (A/broiler duck/Korea/Gochang 1/2014, referred to as the Gochang-like group) were reported among poultry and wild birds in South Korea from January 2014 to July 2015, and subsequently spread to Japan, Russia, Europe, and North America [10]. It was the first incidence of a single AIV subtype resulting in viral transmission over a large geographical area since 2005 [15]. The second wave of H5N8 was detected in 2016. The HPAIV reassortants (represented by A/Bar-headed Goose/Qinghai/BTY1-B/2016 [11] and A/great crested grebe/Uvs-Nuur Lake/341/2016 [12]) with the consensus gene sequences of the Gochang-like group were isolated from dead wild birds from Qinghai Lake on the Tibet–Qinghai Plateau and Uvs-Nuur in Siberia. Thereafter, a wide range of avian species were reported to be affected in 49 countries across Europe, Asia, and Africa [16]. In 2020, a novel H5N8 reassortant generated from Sub-Saharan Africa clade 2.3.4.4b H5N8 HPAIVs and Eurasian LPAIVs was reported to be widely prevalent in wild fowl and domestic poultry in Eurasia and the Middle East. It was considered as the third H5N8 outbreak wave and had a worse impact on poultry than the second wave due to the extent of the spread [13,17].

It has been reported that the seasonal migration of wild aquatic birds may have impacted the transmission and incursion of clade 2.3.4.4 H5N8 HPAIVs globally [1]. H5N8 viruses spread via long-distance migratory flights from the wintering sites of the wild birds to the breeding locations in spring and from breeding locations to wintering sites in the autumn [15]. In China, H5N8 HPAIVs were identified in migrating swans in Hubei and Shandong province in 2020 [18,19]. Consequently, in November 2020, the China National Forestry and Grass Administration issued a document calling for the strengthening of wild bird AIV monitoring systems, and a number of local departments in Yunnan cooperated in carrying out AIV surveillance in Dashanbao Black-Necked Crane National Nature Reserve and Huize Black-Necked Crane National Reserve, in Yunnan Province, China. Four H5N8 isolates from the common crane were identified in this endeavor. Phylogenetic analysis and viral genetic characterization were conducted based on the sequences of eight AIV segments to assess the possible origin of these isolates.

## 2. Materials and Methods

### 2.1. Sample Collection

**Figure 1:**
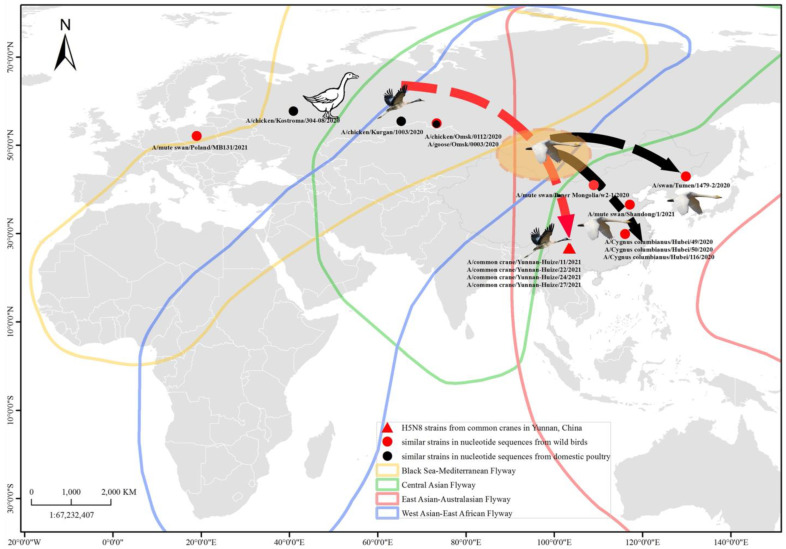
The spatial distribution of H5N8 AIVs isolated in this study and the proposed transmission relationship amongst them and their homology. The red triangle denotes the H5N8 strains identified in this study from common cranes wintering in Yunnan, China. The red and black circles represent the homologous strains from wild birds and domestic poultry, respectively. The red dashed line indicates the overwintering flight route of common cranes from Siberia, Russia, to Yunnan, China; and the black dotted line indicates the overwintering flight route of swans from Mongolia to Hubei, China. The orange-shaded oval area denotes the main stopovers for common cranes and the breeding site for swans. The map was constructed using ArcGIS Desktop version 10.2.

In this surveillance process, 814 fresh fecal samples from wading birds, including 300 common cranes, and 6 environmental water samples were collected in Huize Black-Necked Crane National Nature Reserve and Dashanbao Black-Necked Crane National Nature Reserve, Yunnan Province, China, from November 2020 to March 2021 (Figure 1, Supplementary Table S1).

The species of wild birds were identified by their morphological features as observed through high-power telescopes at their foraging habitats, and the population sizes were recorded. The number of fecal samples collected at each sampling site was not more than 20% of the population of the wild birds, and the distance between each fecal sample was more than 10 m [20]. Environmental water samples from the marsh habitats of the wild birds were also collected. Within 1 h, both the fresh feces and water samples were placed in RNase-free cryogenic vials containing sample preservation solution (pH 7.2 phosphate buffer saline supplemented with 2000 IU/mL penicillin, 2 mg/mL streptomycin, 50 μg/mL gentamicin, and 1000 IU/mL nystatin) and were then transported to the laboratory in white Styrofoam plastic boxes at a low temperature maintained by dry ice.

### 2.2. Genomic Segment Amplification Eight AIV Segments

The collected samples were thawed, vortexed, and centrifuged at 8000 rpm for 10 min, and then the supernatants were taken for viral RNA extraction using a QIAamp viral RNA kit (Qiagen, Hilden, Germany), according to the manufacturer’s instructions. The nucleic acid of five infectious viruses circulating in Yunnan Province, China, including AIV, Newcastle disease virus (NDV), fowl adenovirus serotype 4 (FAdV4), avian infectious bronchitis virus (AIBV), and avian leukemia virus (ALV), were detected. This study focused on HPAIVs. The viral RNA was firstly analyzed by targeting the matrix (*M*) gene of AIV with RT-PCR. Subsequently, the RT-PCR amplification of all eight gene segments of AIV was carried out, and the hemagglutinin (*HA*) and neuraminidase (*NA*) subtypes were identified in AIV *M* gene positive samples.

The RT-PCR primers AIF and AIR for the *M* gene and the primers MBTuni-12 and MBTuni-13 for eight segments of the AIV genome were selected according to the published sequences [20]. The *M* gene and eight segments of the AIV genome were amplified using a PrimeScript One-Step RT-PCR kit (TaKaRa, Kusatsu, Japan) in a 50 μL reaction volume containing 1 μL of enzyme mix, 25 μL (2X) of one-step buffer, 1 μL of each appropriate primer (primers AIF and AIR for the *M* gene or primers MBTuni-12 and MBTuni-13 for the AIV genome), and 2 μL of RNA template. The RT-PCR amplification conditions comprised a cDNA synthesis process at 45 °C for 30 min (*M* gene) or 42 °C for 60 min (AIV genome), 2 min of incubation at 94 °C, and then 35 cycles of amplification as follows: 94 °C denaturation for 30 s, 45 °C (*M* gene) or 57 °C (AIV genome) annealing for 30 s, and 72 °C (*M* gene) or 68 °C (AIV genome) extension for 1 min. *HA* and *NA* subtype detection was conducted using commercial real-time RT-PCR kits (Mabsky, Shenzhen, China) in an ABI7500 system (Applied Biosystems, Foster City, CA, USA), according to the manufacturer’s recommendations.

### 2.3. Next-Generation Sequencing

The RT-PCR products of eight AIV genomic segments (*HA*, *NA*, *M*, polymerase basic (*PB2*), *PB1*, polymerase acidic (*PA*), nucleoprotein (*NP*), nonstructural protein (*NS*)) from the primers MBTuni-12 and MBTuni-13 were further sequenced by next-generation sequencing.

Briefly, genome libraries were generated using the Nextera XT Library Prep Kit (Illumina, CA, USA) and were paired-end sequenced on an Illumina MiSeq platform (Illumina, CA, USA), according to the manufacturer’s instructions. Then, the Fastax online software version 0.0.13 (http://hannonlab.cshl.edu/fastx_toolkit/index.html (accessed on 30 August 2021)) was used to screen out unqualified reads to obtain clean reads. These clean reads were de novo assembled into primary unigenes using CLC Genomics Workbench version 6.0.4, according to the scaffolding contig algorithm (word-size = 45, minimum contig length = 300). Next, the final unigene sequence sets were acquired from primary unigenes via the CAP3 Sequence Assembly Program (http://doua.prabi.fr/software/cap3 (accessed on 30 August 2021)) [21] and were compared against the NCBI nonredundant (Nr) database using BLASTX with an E-value < 10^−5^. The ones annotated as gene sequences of the influenza virus were analyzed.

The AIV positive samples of this study were annotated as H5N8 subtype AIV and their genomic sequences were deposited in the Global Initiative on Sharing All Influenza Data (GISAID) EpiFlu database (https://www.gisaid.org/ (accessed on 21 October 2022)) with the accession numbers of EPI2195573, EPI2195574, EPI2195578-EPI2195580, EPI2195582-EPI2195585, EPI2196441, EPI2195590, EPI2195595-EPI2195598, EPI2195605-EPI2195619, and EPI2195636.

### 2.4. Phylogenetic Analysis and Molecular Characterization

Homology matching was performed for the sequences of the eight segments of H5N8 AIV in this study using the BLAST tool via the GISAID and NCBI database (https://www.ncbi.nlm.nih.gov/ (accessed on 12 April 2022)). The sequences with high similarity to the eight genomic sequences of H5N8 AIVs in this study and the representative strains of clade 2.3.4.4a-h from the previous study [8] were retrieved, as shown in Supplementary Table S2. The nucleotide sequences of each AIV segment were aligned using online MAFFT (Multiple Alignment using Fast Fourier Transform, https://mafft.cbrc.jp/alignment/software/ (accessed on 12 April 2022)) [22]. The maximum likelihood (ML) phylogenetic trees for the eight AIV segments were separately generated from the alignments in the IQ-TREE program version 2.0.3 using the best model of GTR + F + G4 (for PB2), TIM + F + G4 (for PB1 and HA), TIM + F + I (for PA), HKY + F + G4 (for NP), K3Pu + F + I + G4 (for NA), K3P + G4 (for M), and TPM2u + F + G4 (for NS), with the bootstrap values of 1000 replicates [23]. To estimate the time to the most common recent ancestor (tMRCA) for the eight segments of H5N8 AIVs, Bayesian inference was employed using the BEAST package version 1.10.4. The SRD06 nucleotide substitution model and the uncorrelated relaxed clock model with GMRF Bayesian Skyride aggregation model were applied in BEAUti version 1.10.4 to estimate the time-scaled phylogeny for each dataset of the eight genomic segments. A Markov chain Monte Carlo (MCMC) sampling analysis for 100 million iterations with sampling every 1000 steps was run in BEAST version 1.10.4. Three independent runs for each combined model were employed here. The analysis files were combined using LogCombiner version 1.10.4, excluding 10% burn-in to achieve convergence, which was analyzed using Tracer version 1.7.2 with an effective sample size (ESS) greater than 200. The maximum clade creditability (MCC) trees were generated using TreeAnnotator version 1.10.4 and were visualized with FigTree version 1.4.4.

## 3. Results

### 3.1. Virus Determination

Four H5N8 HPAIV isolates, eleven H9N2 isolates, and one of each of NDV, ALV, and FAdV4 were identified in the surveillance process of this study (Supplementary Table S1). The four H5N8 isolates were all from the common crane, and all genomic sequences from eight segments of these isolates were determined, except the deficiency of the sequence of the *M* gene of one H5N8 isolate. These H5N8 AIVs were designated as A/common crane/Yunnan-Huize/11/2021(H5N8) (CC/YH/11/21), A/common crane /Yunnan-Huize/22/2021(H5N8) (CC/YH/22/21), A/common crane/Yunnan-Huize/24/2021(H5N8) (CC/YH/24/21), and A/common crane/Yunnan-Huize/27/2021(H5N8) (CC/YH/27/21).

### 3.2. Phylogenetic Characteristics

Nucleotide homology analysis revealed that the similarity of the whole genomic sequences among the four strains from common cranes in this study ranged from 93.30% to 100.00% (*PB2*, 99.10–100.00%; *PB1*, 98.90–100.00%; *PA*, 93.30–100.00%; *HA*, 98.50–99.40%; *NP*, 99.10–99.90%; *NA*, 97.30–99.80%; *M*, 95.60–100.00%; and *NS*, 95.00–99.50%), indicating that the four isolates are descendants of a common ancestral virus.

According to the BLAST analyses, the eight segments of the four strains from common cranes all shared the highest nucleotide identity with the strains isolated from China, Russia, or Poland in 2020–2021. The nucleotide sequences of all the eight segments of the H5N8 viruses exhibited a sequence identity of 98.46–100% with the H5N8 viruses identified from *Cygnus columbianus* in Hubei Province, China (A/*Cygnus columbianus*/Hubei/49/2020, A/*Cygnus columbianus*/Hubei/50/2020, A/*Cygnus columbianus*/Hubei/51/2020, A/*Cygnus columbianus*/Hubei/52/2020, and A/*Cygnus columbianus*/Hubei/116/2020). The nucleotide sequence identity values of *NP* segments for the four isolates were 97.80–98.72% shared with the H5N8 viruses identified from turkey in Omsk, Siberia, Russia (A/turkey/Omsk/0003/2020). On closer examination, the *HA* gene segments also exhibited a sequence similarity of 96.25–99.20% to the H5N8 viruses isolated from domestic poultry or wild birds in Russia (A/chicken/Omsk/0112/2020, A/chicken/Kostroma/304-08/2020, A/chicken/Kurgan/1003/2020), Poland (A/mute swan/Poland/MB131/2021), and China (A/mute swan/Inner Mongolia/w2-1/2020, A/Mute swan/China/Shangdong1/2021, A/swan/Tumen/1479-2/2020) (Figure 1 and Supplementary Table S3).

**Figure 2:**
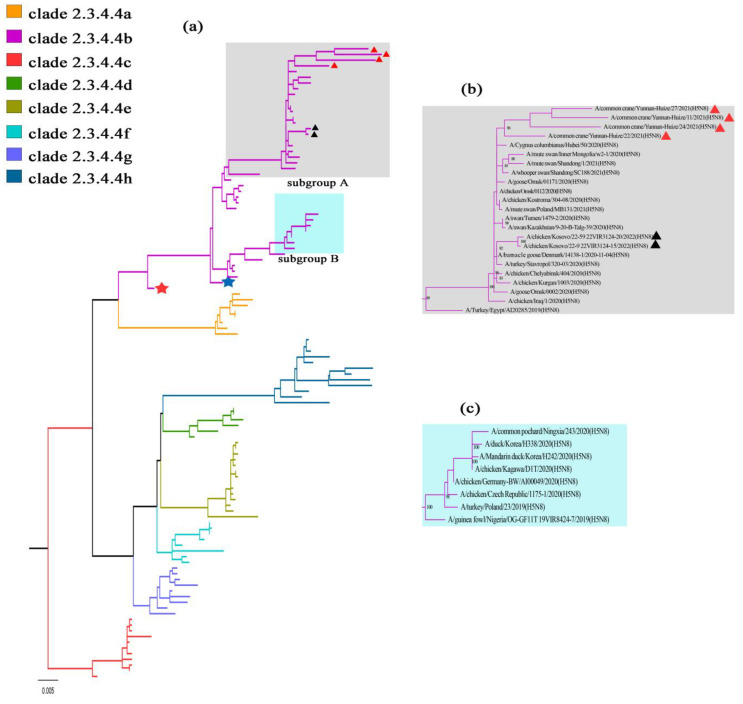
Maximum likelihood (ML) phylogenetic tree of the *HA* gene segment of the H5N8 AIVs. (**a**) Viruses in clade 2.3.4.4a-h are denoted by different colors and two subgroups (subgroup A and B) clustered in clade 2.3.4.4b are marked by different shades of grey and green. The strain marked with a red asterisk was the representative isolation (A/broiler duck/Korea/Gochang1/2014) of the first H5N8 outbreak wave in 2014, and the strain marked with a blue asterisk was the representative isolation (A/Bar-headed Goose/Qinghai/BTY1-B/2016) of the second H5N8 outbreak wave in 2016. (**b**) Subgroup A clusters the H5N8 HPAIVs derived from the ancestral strain of A/Turkey/Egypt/AI20285/2019. The H5N8 HPAIVs from common crane in this study (labelled with a red triangle) and the strains isolated in 2022 (labelled with a black triangle) are clustered in this subgroup. (**c**) Subgroup B clusters the H5N8 HPAIVs derived from the ancestral strain of A/guinea fowl/Nigeria/OG-GF11T_19VIR8724-7. Branch supports were assessed with 1000 bootstrap replicates and only values higher than 70% are shown at the branch nodes.

The ML tree of the *HA* segment demonstrated that four H5N8 viruses from common cranes were derived from the clade 2.3.4.4b H5Nx HPAIVs. All the strains mentioned above were clustered together in this branch with the common ancestral strain of A/broiler duck/Korea/Gochang1/2014 and A/Bar-headed Goose/Qinghai/BTY1-B/2016, which were considered as the representative strains for the first and second global wave of the H5N8 outbreaks in 2014 and 2016, respectively [10,11]. Remarkably, some H5N8 strains isolated in 2022 (such as A/chicken/Kosovo/22-2 22VIR3124-13/2022) were also clustered in this branch (Figure 2a), which indicates that the H5N8 viruses have not changed substantially in terms of phylogenetic characteristics. The clade 2.3.4.4b H5N8 strains identified during 2019–2022 were clustered into two subgroups (subgroups A and B). The viruses in subgroup A were derived from the ancestral strain of A/Turkey/Egypt/AI20285/2019 isolated in Egypt, North Africa, in 2019, while the viruses in subgroup B were from A/guinea fowl/Nigeria/OG-GF11T_19VIR8724-7 (Figure 2b,c). The ML phylogenetic trees of the other seven segments were also derived from the isolate from Egypt, suggesting that the H5N8 HPAIVs from common cranes were standard “Egyptian-like” (Figure S1) [24]. These isolations might show a similar recombination pattern as Egyptian-like H5N8 HPAIVs, with HA, NA, M, and NS gene segments from the Russian HPAI H5N8 virus, and PB2, PB1, PA, and NP segments from the Eurasian H6N2, H5N6, and H1N1 virus [25].

**Figure 3:**
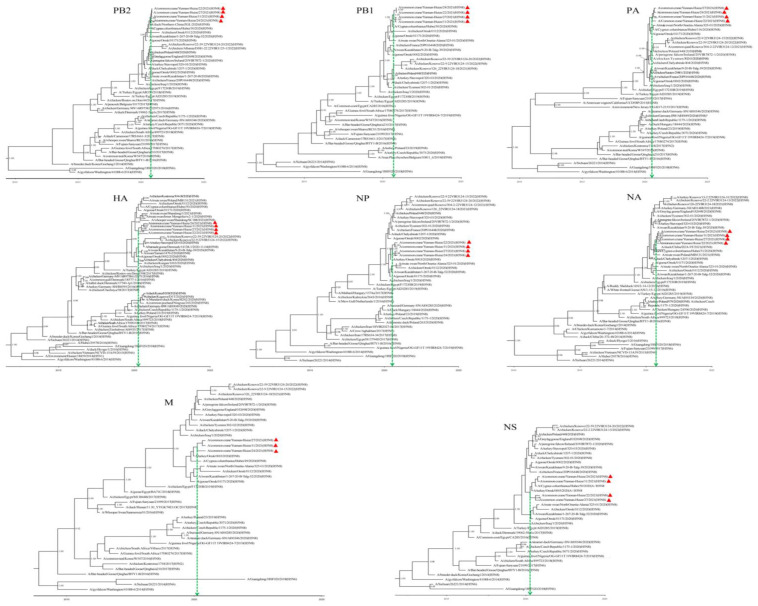
The time-scaled maximum clade credibility (MCC) trees of the eight gene segments of the H5N8 AIVs were constructed using BEAST version 1.10.4. The green dotted vertical lines point indicate the mean time of the most recent common ancestor (tMRCA) of each gene segment. Strains identified in this study are labelled with red triangles. The branch supports are indicated by the Bayesian posterior probabilities and values higher than 0.90 are shown in branch nodes.

The MCC trees of the eight gene segments showed the same genetic topologies with the ML trees (Figure 3 and Figure S1). The timing of the emergence of the strains was estimated in the time-scaled phylogenetic trees by molecular clock analysis. The results indicated that the median tMRCA for the individual gene segments of the H5N8 isolates from common cranes in Yunnan Province, China, ranged from April 2020 (*HA* segment) to October 2020 (*PB2* and *NA* segments), with the 95% credible interval at highest posterior density between January 2019 and November 2020 (Table 1 and Figure 3). This is in agreement with the report that states that the ancestral virus emerged about 5 months before the isolating time of its descendant [26].

### 3.3. Amino Acid Sequence Characteristics

The sequence differences of characteristic amino acids of the eight viral segments were analyzed among H5N8 from common cranes in this study, chicken in Russia (A/chicken/Omsk/0112/2020), *Cygnus columbianus* in China (A/*Cygnus columbianus*/Hubei/50/2020), and the representative strains of clade 2.3.4.4a-h [8] (Supplementary Table S4). In total, 29 amino acid positions were detected. All four H5N8 HPAIVs from common cranes in Yunnan, China, in this study possessed a high-pathogenicity motif of multibasic amino acids (REKRRKR) at the HA cleavage site [27]. In the HA protein, all the isolates sequenced here are characterized by the amino acid substitution of S^137^A, T^160^A, and S^227^R (H3 numbering), which could exhibit the dual-receptor specificity for both human α-2, the 6-linked sialic acid galactose receptor, and the avian α-2, 3-linked sialic acid galactose receptor [5]. The mutation of T^160^A (H3 numbering) results in a lack of a glycosylation site at 158 and facilitates airborne transmission in ferrets [28]. The three polymerases of the H5N8 isolates from common cranes in this study were observed to exhibit L^89^V (in PB2), L^473^V (in PB1), and N^383^D, N^409^S, and S^515^T substitution (in PA), which contribute to enhanced viral replication in mammalian cell lines and serve as markers of mammalian adaptation [29,30,31,32,33]. The phenotype of increased virulence in mice with N^30^D, T^215^A (in M1), P^42^S (in NS), and M^105^V (in NP) mutations were also found in these strains [34,35,36]. Furthermore, the NA protein of the isolates exhibited the same feature as the H5N8 AIV strains circulating in 2020–2021 and contained the long-stalk region, without any amino acid deletion in the neck part of the protein. Although no unique amino acid substitution was detected in the H5N8 HPAIVs from common cranes, the strains and their closest relatives from *Cygnus columbianus* in China and chickens in Russia possess the same NA long stalk as the earlier clade 2.3.4.4a and clade 2.3.4.4c strains (Supplementary Table S4).

## 4. Discussion

The common crane (*Grus grus*) is a migratory wading bird species with an almost worldwide distribution. Its total global population is approximately 491,000–503,000 [37]. The breeding grounds for this species span the entire Eurasian continent, i.e., the regions of northern and western Europe, northern Mongolia, northern China, and eastern Siberia, while the wintering sites include the Mediterranean coast, North and East Africa, the Middle East, India, and southwestern and eastern China [37]. The Huize Black-Necked Crane National Nature Reserve and Dashanbao Black-Necked Crane National Nature Reserve on the Yunnan–Guizhou Plateau serve as the main wintering sites for the common crane in Southwest China [38,39].

In this study, four strains of H5N8 isolates were determined from the feces of common cranes collected in the Huize Black-Necked Crane National Nature Reserve in the spring of 2021 using RT-PCR amplification and AIV whole-genome sequencing. This is the first time that H5N8 HPAIV was identified in the common crane. No wild birds were found dead from clinical diseases in the sampling period, suggesting these H5N8 AIVs were carried by the common cranes asymptomatically.

Phylogenetic analysis of all eight genes revealed that the H5N8 AIVs in the study were clustered together with the H5N8 AIVs isolates of branch 2.3.4.4b from wild birds and poultry in Europe, Russia, Siberia, and China, during 2020–2022 with the common ancestor of A/Turkey/Egypt/AI20285/2019. These viruses presented as standard “Egyptian-like” genetic strains [24]. As an intersection of five global migration routes, including East Asia–Australia, Central Asia, the Black Sea–Mediterranean, West Asia–East Africa, and East Atlantic, Siberia plays a critical role as the breeding ground for wild wading birds from Europe, Africa, the Middle East, Central Asia, and Southeast Asia. The research on the migratory routes of common cranes, based on satellite tracking technology, demonstrates the correlation between common cranes overwintering in Yunnan and breeding in Siberia [40]. In early September, common cranes depart from Siberia, fly through northwestern Mongolia, Inner Mongolia, and Ningxia, China, and arrive to Yunnan for wintering [40] (Figure 1). On the other hand, it has been shown that the swans overwintering in central and northern China migrate from the breeding grounds in north-central and western Mongolia via the Central Asian and the East Asian–Australian Flyway [41]. That is, the migratory swans and common cranes overwintering in China share the same regions as breeding sites and stopover sites, such as Mongolia. Migration activities increase the cross-regional spread of HPAIV, which may account for the similarity of the gene sequences of H5N8 AIV from common cranes in Yunnan Province, the swans in central China, or birds in Siberia. This was supported by a previous study that demonstrated that the H5N8 AIV isolated from swans in central China may have been introduced from Siberia and Europe, because of the close evolutionary relationships among the strains isolated in these regions [19]. “Egyptian-like” H5N8 viruses circulating in Egypt during 2017–2019 were reported to have been disseminated through Iraq into Western Siberia during the spring migration in early 2020 [24]. Then, they might have spread to China during the autumn migration in 2020. As the common crane wintering in Yunnan, China, has a long-distance migration route, in the shared stopover sites during migration, the carried pathogens may be transmitted to other wild bird populations and domestic poultry around due to the high local viral densities in aquatic habitats. Thus, the possibility of viral transmission from common cranes with HPAIV is a matter of concern.

Multiple subtypes of AIVs were reported to have infected various species of mammal due to the ability to overcome mammalian transmission barriers by amino acids mutations [42]. The H5N8 subtype had not been reported to cause human infection until December 2020, when a case of clade 2.3.4.4b H5N8 HPAIV asymptomatic infection of seven farm employees was reported at a poultry farm in Russia [43]. Remarkably, the H5N8 HPAIVs from common cranes possess characteristic amino acid mutations that contribute to a binding preference for the α-2, 6-linked human sialic acid galactose receptor, such as S^128^P (Supplementary Table S4), S^137^A, and S^227^R in HA. These viruses also possess substitutions that contribute to movement from avian to mammalian hosts via increasing the polymerase activity in mammalian cells, such as L^89^V (in PB2), L^473^V (in PB1), and N^383^D, N^409^S, and S^515^T substitution (in PA) [44]. This may suggest that further understanding of viral characterization can provide insight into assessing potential zoonosis outbreaks caused by AIVs.

A short-stalk NA protein with a 19 amino acid deletion in its stalk region was first reported in 1997 [45]. It is known as the molecular marker for AIV adaptation to terrestrial poultry from waterfowl, and will remain deleted during the subsequent transmission [46]. The long stalk in the NA protein of the isolates from common cranes in this study may indicate that the H5N8 AIVs here are derived from wild birds and have not yet become established in domestic poultry.

The continuous spread and the rapid adaptation of clade 2.3.4.4b H5N8 HPAIVs in mammalian hosts should be a serious focus in both research and matters of public health. Wild aquatic birds are the natural host reservoirs for AIV and are responsible for introducing H5N8 AIVs to the previously unaffected areas via long-distance migration. Furthermore, the interspecies transmission of AIVs is possible if the infected wild aquatic birds come into contact with domestic poultry such as chickens and ducks. Hence, it is paramount for countries to monitor AIVs in wild birds, especially wild migratory waterfowl, at stopover sites on their migration routes and their primary habitats, i.e., their breeding and wintering grounds, in order to improve local precautionary measures and prevent AIV zoonosis around the world.

## Figures and Tables

**Table 1:** The mean time of the most recent common ancestor (tMRCA) for the eight genetic segments of H5N8 AIV from common cranes wintering in Yunnan Province, China, in 2021.

Gene Segment ^1^	Mean tMRCA	95% HPD (Highest Posterior Density)	Posterior Probability
*PB2*	October 2020	September 2020, November 2020	1.0000
*PB1*	September 2020	August 2020, October 2020	0.9988
*PA*	July 2020	May 2020, October 2020	1.0000
*HA*	April 2020	November 2019, August 2020	0.9855
*NP*	July 2020	April 2020, September 2020	1.0000
*NA*	October 2020	July 2020, November 2020	0.7117
*MP*	August 2020	June 2020, November 2020	0.7530
*NS*	April 2020	October 2019, June 2020	0.7253

^1^ *PB2*, polymerase basic protein 2; *PB1*, polymerase basic protein 1; *PA*, polymerase acidic protein; *HA*, hemagglutinin; *NP*, nucleoprotein; *NA*, neuraminidase; *MP*, matrix protein; *NS*, nonstructural protein.

## Data Availability

Not applicable.

## Supplementary Materials

The following supporting information can be downloaded at: https://www.mdpi.com/article/10.3390/v15010038/s1, Table S1: The detailed information of the samples in this study; Table S2: The detailed information of the sequences of *HA* gene retrieved from NCBI and GISAID databases used for phylogenetic analysis in this study; Table S3: The nucleotide identity on the eight gene segments of the H5N8 AIVs from common cranes wintering in Yunnan, China, in 2021, with the most similar sequences available in GenBank and GISAID databases; Table S4: The key amino acid substitutions of the four H5N8 AIVs strains from common cranes wintering in Yunnan, China, in 2021 compared with the representative viruses of the clade 2.3.4.4a-h. Figure S1: Maximum likelihood (ML) phylogenetic trees of *PB2* (polymerase basic protein 2), *PB1* (polymerase basic protein 1); *PA* (polymerase acidic protein), *NP* (nucleoprotein), M (matrix), *NS* (nonstructural protein), and *NA* (neuraminidase) gene segments of the H5N8 AIVs.
