# Supplementary material for: Clade 2.3.4.4b H5N8 Subtype Avian Influenza Viruses Were Identified from the Common Crane Wintering in Yunnan Province, China
Source: Viruses. 2022 Dec 22;15(1):38. doi: 10.3390/v15010038 (PMC9863098; doi:10.3390/v15010038)
Supplement: Supplementary file 1 [file viruses-15-00038-s001.zip › viruses-2066256-supplementary.pdf]

Supplementary Table S1. The detailed information of the samples in this study [1].

**Sampling places**	**Wild bird species**	**Sample types**	**Numbers of the sample**	**Numbers of AIV positive sample and the AIV subtypes**	**Numbers of other virus positive samples and the virus type**
Huize Black-necked Crane National Nature Reserve	common crane *Grus grus*	fecal sample	188	4 (H5N8)<br>0 (H9N2)	
	black-necked crane *Grus nigricollis*	fecal sample	217	0 (H5N8)<br>0 (H9N2)	1 (FAdV4)
	bar-headed goose *Anser indicus*	fecal sample	90	0 (H5N8)<br>1 (H9N2)	1 (NDV)
	greylag goose *Anser anser*	fecal sample	6	0 (H5N8)<br>0 (H9N2)	
	unknown species	fecal sample	8	0 (H5N8)<br>0 (H9N2)	
	poultry *Gallus gallus*	cloaca swab sample	104	1 (H5N8)<br>4 (H9N2)	
	environmental water samples	water sample	4	0 (H5N8)<br>0 (H9N2)	
Dashanbao Black-necked Crane National Nature Reserve	common crane *Grus grus*	fecal sample	112	0 (H5N8)<br>4 (H9N2)	
	black-necked crane *Grus nigricollis*	fecal sample	108	0 (H5N8)<br>0 (H9N2)	
	bar-headed goose *Anser indicus*	fecal sample	85	0 (H5N8)<br>1 (H9N2)	1 (ALV)
	poultry *Gallus gallus*	cloaca swab sample	35	0 (H5N8)<br>1 (H9N2)	
	environmental water samples	water sample	2	0 (H5N8)<br>0 (H9N2)	

[1] Nine hundred and fifty-nine fresh fecal samples from wading birds, 139 cloaca swab samples from domestic poultry sharing the common water environment with wild birds and 6 environmental water samples from the marsh habitats of the wild birds were collected in two wetland reserves in Yunnan Province, China, Huize Black-necked Crane National Nature Reserve and Dashanbao Black-necked Crane National Nature Reserve, from November 2020 to March 2021. The nucleic acid of five infectious viruses circulating in Yunnan Province, China, including avian influenza virus (AIV), Newcastle disease virus (NDV), fowl adenovirus serotype 4 (FAdV4), avian infectious bronchitis virus (AIBV) and avian leukemia virus (ALV) were detected. Four strains of H5N8 AIV from common cranes were determined. Additionally, 11 H9N2 isolates and one of each of NDV, ALV and FAdV4 were determined.

Supplementary Table S2. The detailed information of the sequences of *HA* gene retrieved from NCBI and GISAID databases used for phylogenetic analysis in this study.

**Virus strains**	**Accession number**	**Collection date**	**Submitting Laboratory**
A/chicken/Kostroma/304-08/2020(H5N8)	EPI_ISL_1114742	17/10/2020	State Research Center of Virology and Biotechnology (VECTOR)
A/turkey/Stavropol/320-03/2020(H5N8)	EPI_ISL_1114751	11/12/2020	State Research Center of Virology and Biotechnology (VECTOR)
A/chicken/Kosovo/22-9 22VIR3124-15/2022(H5N8)	EPI_ISL_12176852	19/01/2022	Istituto Zooprofilattico Sperimentale delle Venezie
A/chicken/Kosovo/22-59 22VIR3124-20/2022(H5N8)	EPI_ISL_12176855	01/03/2022	Istituto Zooprofilattico Sperimentale delle Venezie
A/chicken/Kagawa/D1T/2020(H5N8)	EPI_ISL_1273424	12/11/2020	National Institute of Animal Health
A/duck/Hebei/2/2011(H5N2)	EPI_ISL_137254	01/12/2011	Institute of Microbiology, Chinese Academy of Sciences
A/breeder duck/Korea/Gochang1/2014(H5N8)	EPI_ISL_157609	01/06/2014	Import from public-domain
A/broiler duck/Korea/Buan2/2014(H5N8)	EPI_ISL_157610	17/01/2014	Import from public-domain
A/Sichuan/26221/2014(H5N6)	EPI_ISL_163493	21/04/2014	WHO Chinese National Influenza Center
A/environment/Zhenjiang/C13/2013(H5N6)	EPI_ISL_167767	20/12/2013	Import from public-domain
A/gyrfalcon/Washington/41088-6/2014(H5N8)	EPI_ISL_173878	08/12/2014	Import from public-domain
A/cat/Sichuan/SC18/2014(H5N6)	EPI_ISL_177761	02/05/2014	Import from public-domain
A/chicken/Tonghai/802/2014(H5N1)	EPI_ISL_179402	12/01/2014	Import from public-domain
A/chicken/Oregon/A01819044/2015(H5N2)	EPI_ISL_179545	11/02/2015	Import from public-domain
A/pigeon/Sichuan/NCXN29/2014(H5N1)	EPI_ISL_179653	27/04/2014	Import from public-domain
A/environment/Jiangsu/12.30 WZNHQ012/2014(H5N6)	EPI_ISL_200258	30/12/2014	Institute of Microbiology, Chinese Academy of Sciences
A/duck/Wuhan/WHYF03/2015(H5N6)	EPI_ISL_205116	01/2015 (Day unknown)	Import from public-domain
A/turtledove/Wuhan/WHBJ12/2014(H5N6)	EPI_ISL_205137	12/2014 (Day unknown)	Import from public-domain
A/chicken/Yunnan/1/2014(H5N1)	EPI_ISL_205856	10/02/2014	Import from public-domain
A/mallard/Oregon/AH0008887/2015(H5N2)	EPI_ISL_206405	07/01/2015	Import from public-domain

Virus strains	Accession number	Collection date	Submitting Laboratory
A/mallard/Washington/196262/2015(H5N2)	EPI_ISL_206436	01/01/2015	Import from public-domain
A/duck/Eastern China/S0908/2014(H5N6)	EPI_ISL_208838	08/09/2014	Import from public-domain
A/duck/Eastern China/S0322/2014 (H5N6)	EPI_ISL_208839	22/03/2014	Import from public-domain
A/mute swan/Poland/MB131/2021(H5N8)	EPI_ISL_2111625	15/02/2021	National Veterinary Research Institute
A/Environment/Hunan/18459/2014(H5N1)	EPI_ISL_219770	24/02/2014	WHO Chinese National Influenza Center
A/Bar-headed Goose/Qinghai/BTY1-B/2016(H5N8)	EPI_ISL_224704	09/05/2016	Wuhan Institute of Virology
A/duck/Vietnam/LBM816/2015(H5N6)	EPI_ISL_231526	10/11/2015	Import from public-domain
A/mallard/Alaska/AH0088535/2016(H5N2)	EPI_ISL_235740	12/08/2016	Import from public-domain
A/turkey/Germany-SH/R8595/2016(H5N8)	EPI_ISL_238037	09/11/2016	Friedrich.Loeffler.Institut
A/Mandarin duck/Korea/K16-187-3/2016(H5N6)	EPI_ISL_238148	28/10/2016	Konkuk University
A/duck/Hyogo/1/2016(H5N6)	EPI_ISL_239351	14/11/2016	National Institute of Infectious Diseases (NIID)
A/teal/Tottori/1/2016(H5N6)	EPI_ISL_243060	15/11/2016	Import from public-domain
A/chicken/Vietnam/NCVD-15A59/2015(H5N6)	EPI_ISL_244518	18/08/2015	Import from public-domain
A/chicken/Hubei/ZYSJF11/2016(H5N6)	EPI_ISL_244528	21/02/2016	Import from public-domain
A/chicken/Hubei/ZYSJF38/2016(H5N6)	EPI_ISL_244531	21/02/2016	Import from public-domain
A/*Cygnus columbianus*/Hubei/50/2020(H5N8)	EPI_ISL_2555543	12/11/2020	Wuhan Institute of Virology, CAS
A/Hubei/29578/2016(H5N6)	EPI_ISL_256213	15/04/2016	WHO Chinese National Influenza Center
A/crane/Kagoshima/KU-27/2016(H5N6)	EPI_ISL_279027	23/11/2016	Kagoshima University
A/common pochard/Ningxia/243/2020(H5N8)	EPI_ISL_2820479	16/10/2020	Northeast Forestry University
A/environment/Niigata/5/2016(H5N6)	EPI_ISL_293486	28/11/2016	Import from public-domain
A/swan/Kazakhstan/9-20-B-Talg-39/2020(H5N8)	EPI_ISL_2932614	21/09/2020	National Center of Biotechnology Republic of Kazakhstan
A/chicken/Yangzhou/YD1/2014(H5N6)	EPI_ISL_295144	01/09/2014	Import from public-domain
A/chicken/Rostov-on-Don/1598/2017(H5N8)	EPI_ISL_297235	17/11/2017	State Research Center of Virology and Biotechnology

Virus strains	Accession number	Collection date	Submitting Laboratory
			(VECTOR)
A/turkey/Wisconsin/15-012886-1/2015(H5N2)	EPI_ISL_300646	20/04/2015	Import from public-domain
A/turkey/Minnesota/15-011666-1/2015(H5N2)	EPI_ISL_301062	11/04/2015	Import from public-domain
A/Guangdong/18SF020/2018(H5N6)	EPI_ISL_337274	29/09/2018	WHO Chinese National Influenza Center
A/chicken/South Africa/17090100/2017(H5N8)	EPI_ISL_369353	06/09/2017	Import from public-domain
A/mallard/Aichi/2312T018/2016(H5N6)	EPI_ISL_372971	13/12/2016	Import from public-domain
A/chicken/Zimbabwe/AI4935/2017(H5N8)	EPI_ISL_387133	25/05/2017	Import from public-domain
A/Turkey/Egypt/AI20285/2019(H5N8)	EPI_ISL_400027	03/04/2019	Friedrich-Loeffler-Institut
A/Greylag goose/Hunan/1/2017(H5N6)	EPI_ISL_400492	08/01/2017	Harbin Veterinary Research Institute (CAAS)
A/turkey/Poland/23/2019(H5N8)	EPI_ISL_402134	30/12/2019	National Veterinary Research Institut Poland, PIWet.PIB
A/duck/Nghe An/5382VTC/2019(H5N6)	EPI_ISL_404208	29/11/2019	State Research Center of Virology and Biotechnology (VECTOR)
A/chicken/Thanh Hoa/13836VTC/2019(H5N6)	EPI_ISL_404988	24/07/2019	State Research Center of Virology and Biotechnology (VECTOR)
A/guinea fowl/Nigeria/OG-GF11T 19VIR8424-7/2019(H5N8)	EPI_ISL_405278	05/07/2019	Istituto Zooprofilattico Sperimentale Delle Venezie
A/chicken/Czech Republic/1175-1/2020(H5N8)	EPI_ISL_405391	17/01/2020	State Veterinary Institute Prague
A/chicken/Zhejiang/13160/2016(H5N6)	EPI_ISL_4071930	10/2016 (Day unknown)	Import from public-domain
A/chicken/Germany-BW/AI00049/2020(H5N8)	EPI_ISL_410291	16/02/2020	Friedrich.Loeffler.Institut
A/chicken/Germany-MV/AR9738-L02971/2016(H5N8)	EPI_ISL_436136	22/11/2016	Friedrich.Loeffler.Institut
A/duck/Ha Tinh/HT12/2014(H5N6)	EPI_ISL_504005	14/05/2014	Import from public-domain
A/chicken/South Africa/499723/2018(H5N8)	EPI_ISL_505432	06/06/2018	Import from public-domain
A/mute swan/Shandong/1/2021(H5N8)	EPI_ISL_5260453	14/01/2021	Harbin Veterinary Research Institute (CAAS)

Virus strains	Accession number	Collection date	Submitting Laboratory
A/whooper swan/Shandong/SC188/2021(H5N8)	EPI_ISL_5260461	22/02/2021	Harbin Veterinary Research Institute (CAAS)
A/chicken/Iraq/1/2020(H5N8)	EPI_ISL_623074	12/05/2020	Animal and Plant Health Agency (APHA)
A/mute swan/Inner Mongolia/w2-1/2020(H5N8)	EPI_ISL_625672	17/10/2020	Northeast Forestry University
A/Mandarin duck/Korea/H242/2020(H5N8)	EPI_ISL_631824	21/10/2020	Animal and Plant Quarantine Agency (S.2158)
A/goose/Omsk/0002/2020(H5N8)	EPI_ISL_644122	13/08/2020	State Research Center of Virology and Biotechnology (VECTOR)
A/goose/Omsk/01171/2020(H5N8)	EPI_ISL_644132	17/08/2020	State Research Center of Virology and Biotechnology (VECTOR)
A/chicken/Omsk/0112/2020(H5N8)	EPI_ISL_644150	17/08/2021	State Research Center of Virology and Biotechnology (VECTOR)
A/chicken/Chelyabinsk/404/2020(H5N8)	EPI_ISL_644161	06/08/2020	State Research Center of Virology and Biotechnology (VECTOR)
A/barnacle goose/Denmark/14138-1/2020-11-04(H5N8)	EPI_ISL_644824	04/11/2020	Statens Serum Institute
A/chicken/Kurgan/1003/2020(H5N8)	EPI_ISL_654835	27/08/2020	State Research Center of Virology and Biotechnology (VECTOR)
A/chicken/Thanh Hoa/844VTC/2020(H5N6)	EPI_ISL_654849	18/02/2020	State Research Center of Virology and Biotechnology (VECTOR)
A/chicken/Fujian/9.24 FZHX0076-O/2018(H5)	EPI_ISL_697989	24/09/2018	Import from public-domain
A/chicken/Thanh Hoa/1152VTC/2020(H5N6)	EPI_ISL_654859	06/03/2020	State Research Center of Virology and Biotechnology (VECTOR)
A/chicken/Poland/448/2020(H5N8)	EPI_ISL_661177	24/11/2020	National Veterinary Research Institut Poland, PIWet-PIB
A/swan/Tumen/1479-2/2020(H5N8)	EPI_ISL_661178	10/09/2020	Federal Centre for Animal Health (ARRIAH)
A/chicken/France/20P016448/2020(H5N8)	EPI_ISL_667810	10/11/2020	ANSES Agence Nationale De Securite Sanitaire De

Virus strains	Accession number	Collection date	Submitting Laboratory
			L'alimentation
A/goose/Fujian/3.15 FZHX0007-C/2018(H5N6)	EPI_ISL_696995	15/03/2018	Import from public-domain
A/duck/Zhejiang/12.28 HZBX015-O/2018(mixed)	EPI_ISL_697912	28/12/2018	Import from public-domain
A/chicken/Hunan/12.24 YYGK35E4-O/2018(H5N6)	EPI_ISL_698066	24/12/2018	Import from public-domain
A/Whooper swan/Mongolia/25/2020(H5N6)	EPI_ISL_707016	30/04/2020	Import from public-domain
A/duck/Hunan/1.12 YYGK68H3-OC/2018(H5N6)	EPI_ISL_707489	12/01/2018	Import from public-domain
A/duck/Hunan/1.12 YYGK72H3-OC/2018(H5N6)	EPI_ISL_707490	12/01/2018	Import from public-domain
A/Greylag goose/England/032698/2020(H5N8)	EPI_ISL_710507	03/11/2020	Animal and Plant Health Agency (APHA)
A/peregrine falcon/Ireland/20VIR7872-1/2020(H5N8)	EPI_ISL_813979	11/2020 (Day unknown)	Istituto Zooprofilattico Sperimentale Delle Venezie
A/duck/Korea/H338/2020(H5N8)	EPI_ISL_985179	26/11/2020	Animal and Plant Quarantine Agency (S.2158)
A/wild bird/Jiangxi/P410/2015(H5N1)	KX960166.1	07/01/2015	China Animal Health and Epidemiology Center, Laboratory of Avian Disease Surveillance
A/wild bird/Jiangxi/P5/2015(H5N6)	KX960176.1	07/01/2015	China Animal Health and Epidemiology Center, Laboratory of Avian Disease Surveillance
A/chicken/Anhui/MZ34/2016(H5N6)	KY005860.1	02/2016 (Day unknown)	Yangzhou University
A/chicken/Anhui/QD1/2014(H5N1)	KY437807.1	27/02/2014	Yangzhou University，Animal Infectious Disease Laboratory, College of Veterinary Medicine
A/American wigeon/California/UCD58P/2015(H5N8)	KY828643.1	17/01/2015	Center for Research on Influenza Pathogenesis (CRIP), New York
A/bean goose/Ishikawa/1701A012/2017(H5N6)	LC275034.1	06/12/2017	Kosuke Soda Tottori University
A/muscovy duck/Vietnam/LBM817/2015(H5N6)	LC279787.1	10/11/2015	Kosuke Soda Tottori University, Avian Zoonosis Research Center, Faculty of Agriculture
A/chicken/Nha Trang/122/2015(H5N6)	LC279821.1	14/11/2015	Kosuke Soda Tottori University

Virus strains	Accession number	Collection date	Submitting Laboratory
A/northern goshawk/Tochigi/0912A004/2016(H5N6)	LC306911.1	12/12/2016	Kosuke Soda Tottori University
A/northern goshawk/Tochigi/0912A004/2016(H5N6)	LC306911.1	12/12/2016	Kosuke Soda Tottori University
A/tundra swan/Niigata/5112006/2016(H5N6)	LC316696.1	04/12/2016	Kosuke Soda Tottori University, Avian Zoonosis Research Center, Faculty of Agriculture
A/whooper swan/Hokkaido/X13/2017(H5N6)	LC318461.1	18/01/2017	Makiko Jizou Hokkaido University, Faculty of Veterinary Medicine
A/tundra swan/Iwate/9/2016(H5N6)	LC318829.1	22/12/2016	Makiko Jizou Hokkaido University, Faculty of Veterinary Medicine
A/water/Aichi/C3/2016(H5N6)	LC348831.1	17/12/2016	Kosuke Soda Tottori University, Avian Zoonosis Research Center, Faculty of Agriculture
A/chicken/Chechnya/58/2017(H5N8)	MF926461.1	08/01/2017	GamaleyaNational Research Centre for Epidemiology and Microbiology
A/environment/Chang Sha/399/2014(H5N6)	MH156491.1	18/09/2014	Changsha Center for Disease Control and Prevention
A/Guinea fowl/South Africa/17080274/2017(H5N8)	MH165628.1	16/08/2017	Faculty of Veterinary Science, University of Pretoria
A/duck/Viet Nam/HN-2431/2015(H5N1)	MK943249.1	10/01/2015	Craig Venter Institute
A/common gull/Denmark/18577-1/2016(H5N8)	MW026088.1	16/11/2016	Statens Serum Institut
A/tufted duck/Denmark/17740-1p1/2016(H5N8)	MW026128.1	07/11/2016	Statens Serum Institut

Supplementary Table S3. The nucleotide identity on the eight gene segments of the H5N8 AIVs from common cranes wintering in Yunnan, China in 2021, with the most similar sequences available in GenBank and GISAID databases.

**Virus strains** [1]	**Gene segment** [2]	**Accession number**	**Similar virus strains in databases**	**Nucleotide identity (%)**
CC/YH/11/21	*PB2*	MW505390.1	A/*Cygnus columbianus*/Hubei/52/2020(H5N8)	99.76
		EPI1844088	A/duck/Northern China/ZGL/2020(H5N8)	99.61
	PB1	EPI1873113	A/*Cygnus columbianus*/Hubei/50/2020 (H5N8)	99.31
	PA	EPI1873066	A/*Cygnus columbianus*/Hubei/116/2020 (H5N8)	99.03
	HA	MW505375.1	A/*Cygnus columbianus*/Hubei/50/2020(H5N8)	99.71
		EPI1813345	A/chicken/Omsk/0112/2020 (H5N8)	98.80
		EPI1848654	A/chicken/Kostroma/304-08/2020 (H5N8)	98.75
		EPI1814684	A/swan/Tumen/1479-2/2020 (H5N8)	98.52
		EPI1859647	A/mute swan/Poland/MB131/2021 (H5N8)	98.46
		EPI1814353	A/chicken/Kurgan/1003/2020 (H5N8)	98.41
		EPI1811652	A/mute swan/Inner Mongolia/w2-1/2020 (H5N8)	96.41
		EPI1921518	A/Mute swan/China/Shangdong1/2021 (H5N8	96.41
	NP	MW505378.1	A/*Cygnus columbianus*/Hubei/50/2020(H5N8)	99.87
		EPI1813122	A/turkey/Omsk/0003/2020 (H5N8)	98.16
	NA	EPI1873109	A/*Cygnus columbianus*/Hubei/51/2020 (H5N8)	99.66
	M	EPI1873126	A/*Cygnus columbianus*/Hubei/49/2020 (H5N8)	99.90
	NS	EPI1873119	A/*Cygnus columbianus*/Hubei/50/2020 (H5N8)	100.00
CC/YH/22/21	*PB2*	EPI1873112	A/*Cygnus columbianus*/Hubei/50/2020 (H5N8)	98.46
	PB1	EPI1873113	A/*Cygnus columbianus*/Hubei/50/2020 (H5N8)	99.83
	PA	EPI1873066	A/*Cygnus columbianus*/Hubei/116/2020 (H5N8)	99.68
	HA	MW505375.1	A/*Cygnus columbianus*/Hubei/50/2020(H5N8)	99.72
		EPI1813345	A/chicken/Omsk/0112/2020 (H5N8)	99.20
		EPI1848654	A/chicken/Kostroma/304-08/2020 (H5N8)	99.15
		EPI1814353	A/chicken/Kurgan/1003/2020 (H5N8)	98.81
		EPI1814684	A/swan/Tumen/1479-2/2020 (H5N8)	98.35
		EPI1859647	A/mute swan/Poland/MB131/2021 (H5N8)	98.29
		EPI1811652	A/mute swan/Inner Mongolia/w2-1/2020 (H5N8)	96.25
		EPI1921518	A/Mute swan/China/Shangdong1/2021 (H5N8	96.25
	NP	MW505378.1	A/*Cygnus columbianus*/Hubei/50/2020(H5N8)	99.87
		EPI1813122	A/turkey/Omsk/0003/2020 (H5N8)	98.41
	NA	EPI1873109	A/*Cygnus columbianus*/Hubei/51/2020 (H5N8)	99.59
	NS	EPI1873119	A/*Cygnus columbianus*/Hubei/50/2020 (H5N8)	99.04
CC/YH/24/21	*PB2*	EPI1873112	A/*Cygnus columbianus*/Hubei/50/2020 (H5N8)	99.79
	PB1	EPI1873113	A/*Cygnus columbianus*/Hubei/50/2020 (H5N8)	99.78
	PA	EPI1873066	A/*Cygnus columbianus*/Hubei/116/2020 (H5N8)	99.68
	HA	MW505375.1	A/*Cygnus columbianus*/Hubei/50/2020(H5N8)	99.71
		EPI1813345	A/chicken/Omsk/0112/2020 (H5N8)	98.46
		EPI1848654	A/chicken/Kostroma/304-08/2020 (H5N8)	98.40
		EPI1814684	A/swan/Tumen/1479-2/2020 (H5N8)	98.06

Virus strains [1]	Gene segment [2]	Accession number	Similar virus strains in databases	Nucleotide identity (%)
		EPI1859647	A/mute swan/Poland/MB131/2021 (H5N8)	98.06
		EPI1814353	A/chicken/Kurgan/1003/2020 (H5N8)	98.06
		EPI1811652	A/mute swan/Inner Mongolia/w2-1/2020 (H5N8)	96.63
		EPI1921518	A/Mute swan/China/Shangdong1/2021 (H5N8	96.63
	NP	MW505378.1	A/*Cygnus columbianus*/Hubei/50/2020(H5N8)	99.87
		EPI1813122	A/turkey/Omsk/0003/2020 (H5N8)	98.72
	NA	EPI1873109	A/*Cygnus columbianus*/Hubei/51/2020 (H5N8)	99.72
	M	EPI1873126	A/*Cygnus columbianus*/Hubei/49/2020 (H5N8)	99.61
	NS	EPI1873119	A/*Cygnus columbianus*/Hubei/50/2020 (H5N8)	100.00
CC/YH/27/21	*PB2*	EPI1873112	A/*Cygnus columbianus*/Hubei/50/2020 (H5N8)	99.78
	PB1	EPI1873113	A/*Cygnus columbianus*/Hubei/50/2020 (H5N8)	99.78
	PA	EPI1873066	A/*Cygnus columbianus*/Hubei/116/2020 (H5N8)	99.73
	HA	MW505375.1	A/*Cygnus columbianus*/Hubei/50/2020(H5N8)	99.71
		EPI1813345	A/chicken/Omsk/0112/2020 (H5N8)	99.09
		EPI1848654	A/chicken/Kostroma/304-08/2020 (H5N8)	99.03
		EPI1814684	A/swan/Tumen/1479-2/2020 (H5N8)	98.52
		EPI1859647	A/mute swan/Poland/MB131/2021 (H5N8)	98.46
		EPI1814353	A/chicken/Kurgan/1003/2020 (H5N8)	98.69
		EPI1811652	A/mute swan/Inner Mongolia/w2-1/2020 (H5N8)	96.41
		EPI1921518	A/Mute swan/China/Shangdong1/2021 (H5N8	96.41
	NP	MW505378.1	A/*Cygnus columbianus*/Hubei/50/2020(H5N8)	99.87
		EPI1813122	A/turkey/Omsk/0003/2020 (H5N8)	97.80
	NA	EPI1873109	A/*Cygnus columbianus*/Hubei/51/2020 (H5N8)	99.59
	M	MW505408.1	A/*Cygnus columbianus*/Hubei/56/2020(H5N8)	99.80
		EPI1813124	A/turkey/Omsk/0003/2020 (H5N8)	95.60
	NS	EPI1873119	A/*Cygnus columbianus*/Hubei/50/2020 (H5N8)	100.00

[1] Four viruses identified in this study. CC/YH/27/21: A/common crane/Yunnan-Huize/11/2021(H5N8); CC/YH/22/21: A/common crane /Yunnan-Huize/22/2021(H5N8); CC/YH/24/21: A/common crane/Yunnan-Huize/24/2021(H5N8); CC/YH/27/21: A/common crane/Yunnan-Huize/27/2021(H5N8).

[2] *PB2*, polymerase basic protein 2; *PB1*, polymerase basic protein 1; *PA*, polymerase acidic protein; *HA*, hemagglutinin; *NP*, nucleoprotein; *NA*, neuraminidase; *MP*, matrix protein; *NS*, nonstructural protein.

Supplementary Table S4. The key amino acid substitutions of the four H5N8 AIVs strains from common cranes wintering in Yunnan, China in 2021 compared with the representative viruses of the clade 2.3.4.4a-h.

			**Year of isolation**									
			**2014**	**2014**	**2015**	**2016**	**2016**	**2017**	**2018**	**2019**	**2020**	**2021**
			**Evolutionary clade of *HA* [1]**									
			**2.3.4.4a**	**2.3.4.4c**	**2.3.4.4f**	**2.3.4.4d**	**2.3.4.4e**	**2.3.4.4b**	**2.3.4.4h**	**2.3.4.4g**	**2.3.4.4b**	**2.3.4.4b**
			**Virus strains [2]**									
			**Sichuan/26221**	**Gyrfalcon/ WHT/41088-6**	**CK/VN/NCVD-15A59**	**Hubei/29578**	**DK/hyogo/1**	**Fujian/21099**	**GD/18SF020**	**CK/DN/25437VTC**	**CK/OS/0112, CC/HB/50**	**CC/YH/11/21 CC/YH/22/21 CC/YH/24/21 CC/YH/2721**
**Protein**	**Phenotype**	**Amino acid position**	**Amino acid substitution**									
HA	HPAIV molecular characteristics	Cleavage site [3]	REKRRKR	RERRRKE	RERRRKE	RERRRKE	RERRRKE	REKRRKR	RERRRKE	RERRRKE	REKRRKE	REKRRKR
	Lack of Glycosylation site to facilitate airborne transmission in ferrets and binding preference for the human α-2, 6-linked sialic acid galactose receptor	$N^{158}D$	N	N	N	N	N	N	N	N	N	N
		$T^{160}A$	A	A	A	A	A	A	A	A	A	A
	Binding preference for the human α-2, 6-linked sialic acid galactose receptor	$S^{128}P$	T	P	P	P	P	P	S	P	P	P
		$S^{137}A$	A	A	A	A	A	A	A	A	A	A
		$Q^{226}L$	Q	Q	Q	Q	Q	Q	Q	Q	Q	Q
		$S^{227}R$	R	S	R	S	Q	R	R	R	R	R
		$G^{228}S$	G	G	G	G	G	G	G	G	G	G
NA	Adaptation to terrestrial poultry	Stalk deletions [4]	No	No	Yes	Yes	Yes	Yes	Yes	Yes	No	No
	Oseltamivir and zanamivir resisitance	$I^{117}T$	T	I	T	T	T	T	T	T	I	I
		$H^{274}Y$	H	H	H	H	H	H	H	H	H	H
		$N^{294}S$	N	N	N	N	N	N	N	N	N	N
PB2	Increased polymerase activity and replication in mammalian cell lines	$L^{89}V$	V	V	V	V	V	V	V	I	V	V
		$E^{627}K$	E	E	E	E	E	E	X	E	E	E
		$D^{701}N$	N	D	D	D	D	D	D	D	D	D
PB1	Increased polymerase activity and replication in mammalian cell lines	$L^{473}V$	V	V	V	V	V	V	V	V	V	V
		$L^{598}P$	L	L	L	L	L	L	L	L	L	L
PA	Increased polymerase	$N^{383}D$	D	D	D	D	D	D	D	D	D	D

			Year of isolation									
			**2014**	**2014**	**2015**	**2016**	**2016**	**2017**	**2018**	**2019**	**2020**	**2021**
			**Evolutionary clade of *HA* [1]**									
			**2.3.4.4a**	**2.3.4.4c**	**2.3.4.4f**	**2.3.4.4d**	**2.3.4.4e**	**2.3.4.4b**	**2.3.4.4h**	**2.3.4.4g**	**2.3.4.4b**	**2.3.4.4b**
			**Virus strains [2]**									
			**Sichuan/26221**	**Gyrfalcon/ WHT/41088-6**	**CK/VN/NCVD-15A59**	**Hubei/29578**	**DK/hyogo/1**	**Fujian/21099**	**GD/18SF020**	**CK/DN/25437VTC**	**CK/OS/0112, CC/HB/50**	**CC/YH/11/21 CC/YH/22/21 CC/YH/24/21 CC/YH/2721**
**Protein**	**Phenotype**	**Amino acid position**	**Amino acid substitution**									
	activity and replication in duck and mammalian cell lines	N^{409}S	S	S	N	S	S	S	S	S	S	S
		S^{515}T	T	T	T	T	T	T	T	T	T	T
NP	Increased pathogenicity in chickens	M^{105}V	M	M	I	V	I	I	I	I	V	V
		I^{109}T	I	I	I	I	I	I	I	I	I	I
M1	Increased pathogenicity in mice	N^{30}D	D	D	D	D	D	D	D	D	D	D
		I^{43}M	M	M	M	M	M	M	M	M	M	M
		T^{215}A	A	A	A	A	A	A	A	A	A	A
M2	Amantadine and rimantadine resistance	L^{26}F	L	L	L	L	L	L	L	L	L	L
		S^{31}N	S	N	S	N	S	S	S	S	S	S
NS	Increased pathogenicity in mice	P^{42}S	S	S	S	S	S	S	S	S	S	S
	Increased pathgenicity in chickens	80-84 deletion	Yes	No	Yes	No	Yes	No	Yes	Yes	No	No

[1] The clades were defined referring to the report by World Health Organization.

[2] Sichuan/26221, A/Sichuan/26221/2014(H5N6); Gyrfalcon/WHT/41088-6, A/gyrfalcon/Washington/41088-6/2014(H5N8); CK/VN/NCVD-15A59, A/chicken/VietNam/NCVD-15A59/2015(H5N6); Hubei/29578, A/Hubei/29578/2016(H5N6); DK/hyogo/1, A/duck/Hyogo/1/2016(H5N6); Fujian/21099, A/Fujian-Sanyuan/21099/2017(H5N6); GD/18SF020, A/Guangdong/18SF020/2018(H5N6); CK/DN/25437VTC, A/chicken/Dong Nai/25437VTC/2019(H5N6); CK/OS/0112, A/chicken/Omsk/0112/2020(H5N8); CC/HB/50, A/*Cygnus columbianus*/Hubei/50/2020(H5N8); CC/YH/11/21, A/common crane/Yunnan-Huize/11/2021(H5N8); CC/YH/22/21, A/common crane/Yunnan-Huize/22/2021(H5N8); CC/YH/24/21, A/common crane/Yunnan-Huize/24/2021(H5N8); CC/YH/27/21, A/common crane/Yunnan-Huize/27/2021(H5N8).

[3] At the amino acid positions of 339-345 (H3 numbering).

[4] At the amino acid positions of 58-68 (N8 numbering).

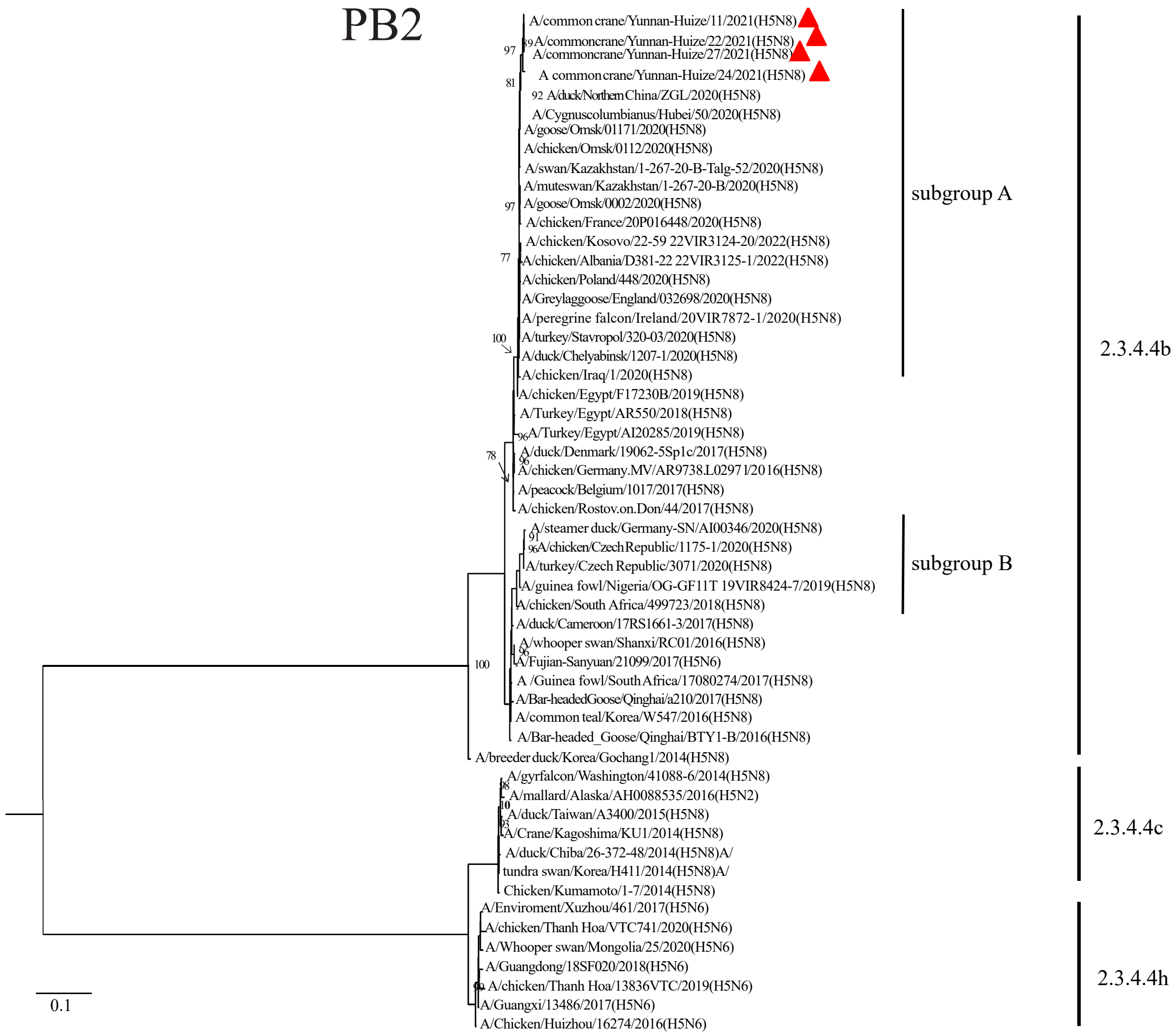
PB2
A/common crane/Yunnan-Huize/11/2021(H5N8)
A/commoncrane/Yunnan-Huize/22/2021(H5N8)
A/commoncrane/Yunnan-Huize/27/2021(H5N8)
A common crane/Yunnan-Huize/24/2021(H5N8)
A/duck/Northern China/ZGL/2020(H5N8)
A/Cygnuscolumbianus/Hubei/50/2020(H5N8)
A/goose/Omsk/01171/2020(H5N8)
A/chicken/Omsk/0112/2020(H5N8)
A/swan/Kazakhstan/1-267-20-B-Talg-52/2020(H5N8)
A/muteswan/Kazakhstan/1-267-20-B/2020(H5N8)
A/goose/Omsk/0002/2020(H5N8)
A/chicken/France/20P016448/2020(H5N8)
A/chicken/Kosovo/22-59 22VIR3124-20/2022(H5N8)
A/chicken/Albania/D381-22 22VIR3125-1/2022(H5N8)
A/chicken/Poland/448/2020(H5N8)
A/Greylaggoose/England/032698/2020(H5N8)
A/peregrine falcon/Ireland/20VIR7872-1/2020(H5N8)
A/turkey/Stavropol/320-03/2020(H5N8)
A/duck/Chelyabinsk/1207-1/2020(H5N8)
A/chicken/Iraq/1/2020(H5N8)
A/chicken/Egypt/F17230B/2019(H5N8)
A/Turkey/Egypt/AR550/2018(H5N8)
A/Turkey/Egypt/AI20285/2019(H5N8)
A/duck/Denmark/19062-5Sp1c/2017(H5N8)
A/chicken/Germany.MV/AR9738.L02971/2016(H5N8)
A/peacock/Belgium/1017/2017(H5N8)
A/chicken/Rostov.on.Don/44/2017(H5N8)
A/steamer duck/Germany-SN/AI00346/2020(H5N8)
A/chicken/Czech Republic/1175-1/2020(H5N8)
A/turkey/Czech Republic/3071/2020(H5N8)
A/guinea fowl/Nigeria/OG-GF11T 19VIR8424-7/2019(H5N8)
A/chicken/South Africa/499723/2018(H5N8)
A/duck/Cameroon/17RS1661-3/2017(H5N8)
A/whooper swan/Shanxi/RC01/2016(H5N8)
A/Fujian-Sanyuan/21099/2017(H5N6)
A /Guinea fowl/South Africa/17080274/2017(H5N8)
A/Bar-headedGoose/Qinghai/a210/2017(H5N8)
A/common teal/Korea/W547/2016(H5N8)
A/Bar-headed_Goose/Qinghai/BTY1-B/2016(H5N8)
A/breeder duck/Korea/Gochang1/2014(H5N8)
A/gyrfalcon/Washington/41088-6/2014(H5N8)
A/mallard/Alaska/AH0088535/2016(H5N2)
A/duck/Taiwan/A3400/2015(H5N8)
A/Crane/Kagoshima/KU1/2014(H5N8)
A/duck/Chiba/26-372-48/2014(H5N8)A/
tundra swan/Korea/H411/2014(H5N8)A/
Chicken/Kumamoto/1-7/2014(H5N8)
A/Enviroment/Xuzhou/461/2017(H5N6)
A/chicken/Thanh Hoa/VTC741/2020(H5N6)
A/Whooper swan/Mongolia/25/2020(H5N6)
A/Guangdong/18SF020/2018(H5N6)
A/chicken/Thanh Hoa/13836VTC/2019(H5N6)
A/Guangxi/13486/2017(H5N6)
A/Chicken/Huizhou/16274/2016(H5N6)
subgroup A
subgroup B
2.3.4.4b
2.3.4.4c
2.3.4.4h
0.1

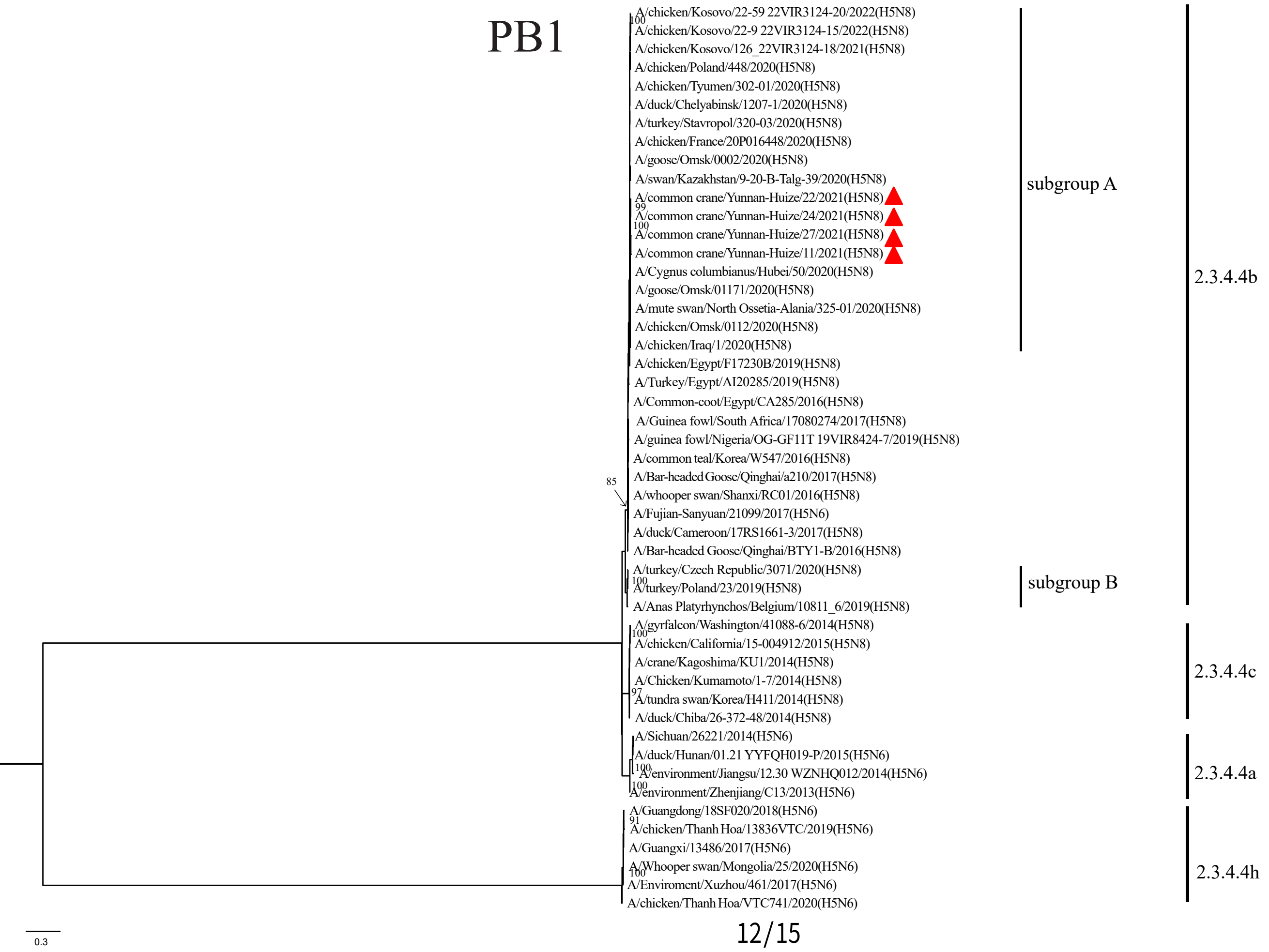
PB1
A/chicken/Kosovo/22-59 22VIR3124-20/2022(H5N8)
A/chicken/Kosovo/22-9 22VIR3124-15/2022(H5N8)
A/chicken/Kosovo/126_22VIR3124-18/2021(H5N8)
A/chicken/Poland/448/2020(H5N8)
A/chicken/Tyumen/302-01/2020(H5N8)
A/duck/Chelyabinsk/1207-1/2020(H5N8)
A/turkey/Stavropol/320-03/2020(H5N8)
A/chicken/France/20P016448/2020(H5N8)
A/goose/Omsk/0002/2020(H5N8)
A/swan/Kazakhstan/9-20-B-Talg-39/2020(H5N8)
A/common crane/Yunnan-Huize/22/2021(H5N8)
A/common crane/Yunnan-Huize/24/2021(H5N8)
A/common crane/Yunnan-Huize/27/2021(H5N8)
A/common crane/Yunnan-Huize/11/2021(H5N8)
A/Cygnus columbianus/Hubei/50/2020(H5N8)
A/goose/Omsk/01171/2020(H5N8)
A/mute swan/North Ossetia-Alania/325-01/2020(H5N8)
A/chicken/Omsk/0112/2020(H5N8)
A/chicken/Iraq/1/2020(H5N8)
A/chicken/Egypt/F17230B/2019(H5N8)
A/Turkey/Egypt/AI20285/2019(H5N8)
A/Common-coot/Egypt/CA285/2016(H5N8)
A/Guinea fowl/South Africa/17080274/2017(H5N8)
A/guinea fowl/Nigeria/OG-GF11T 19VIR8424-7/2019(H5N8)
A/common teal/Korea/W547/2016(H5N8)
A/Bar-headed Goose/Qinghai/a210/2017(H5N8)
A/whooper swan/Shanxi/RC01/2016(H5N8)
A/Fujian-Sanyuan/21099/2017(H5N6)
A/duck/Cameroon/17RS1661-3/2017(H5N8)
A/Bar-headed Goose/Qinghai/BTY1-B/2016(H5N8)
A/turkey/Czech Republic/3071/2020(H5N8)
A/turkey/Poland/23/2019(H5N8)
A/Anas Platyrhynchos/Belgium/10811_6/2019(H5N8)
A/gyrfalcon/Washington/41088-6/2014(H5N8)
A/chicken/California/15-004912/2015(H5N8)
A/crane/Kagoshima/KU1/2014(H5N8)
A/Chicken/Kumamoto/1-7/2014(H5N8)
A/tundra swan/Korea/H411/2014(H5N8)
A/duck/Chiba/26-372-48/2014(H5N8)
A/Sichuan/26221/2014(H5N6)
A/duck/Hunan/01.21 YYFQH019-P/2015(H5N6)
A/environment/Jiangsu/12.30 WZNHQ012/2014(H5N6)
A/environment/Zhenjiang/C13/2013(H5N6)
A/Guangdong/18SF020/2018(H5N6)
A/chicken/Thanh Hoa/13836VTC/2019(H5N6)
A/Guangxi/13486/2017(H5N6)
A/Whooper swan/Mongolia/25/2020(H5N6)
A/Enviroment/Xuzhou/461/2017(H5N6)
A/chicken/Thanh Hoa/VTC741/2020(H5N6)
subgroup A
subgroup B
2.3.4.4b
2.3.4.4c
2.3.4.4a
2.3.4.4h
0.3

cont.

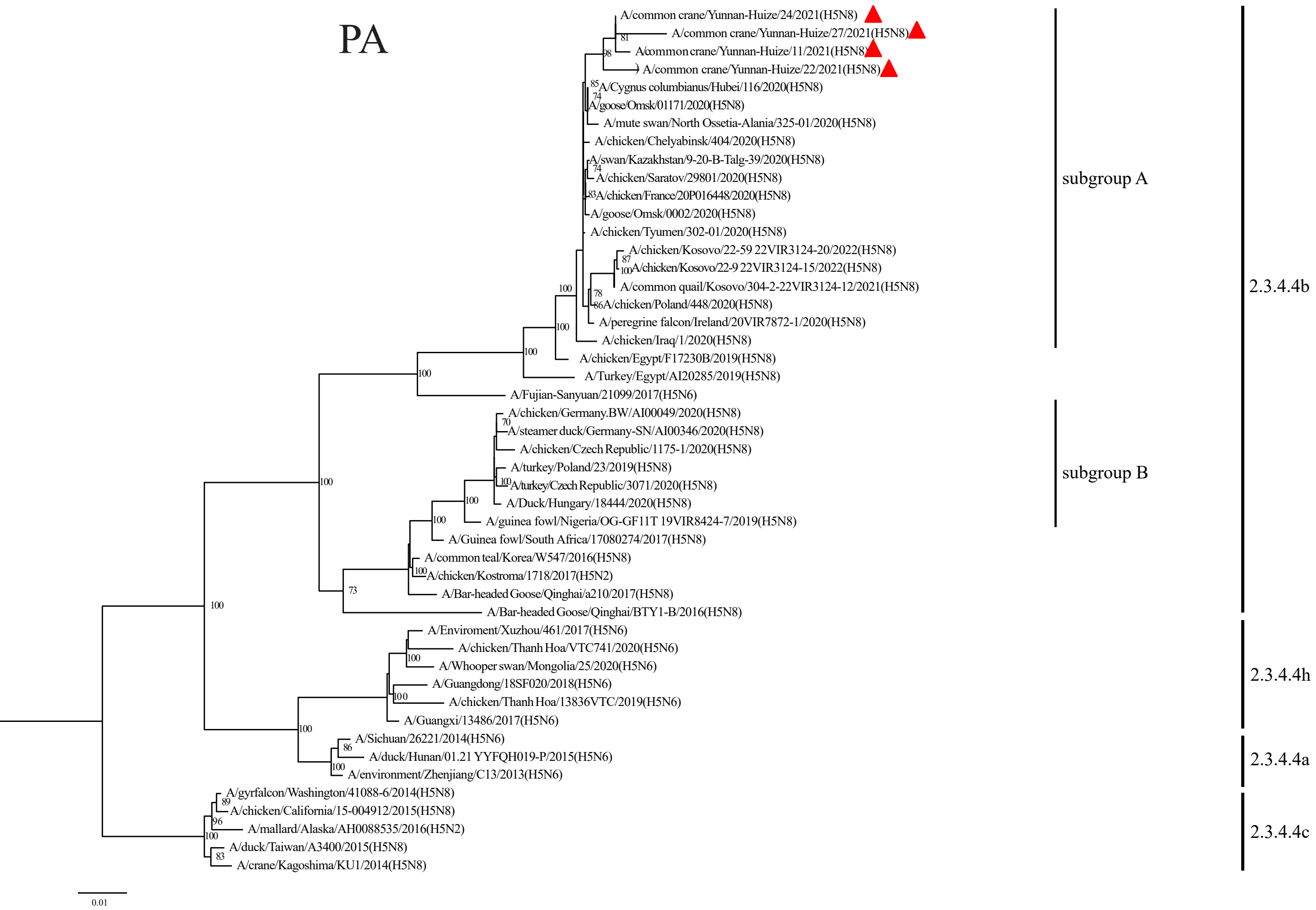
PA
A/common crane/Yunnan-Huize/24/2021(H5N8)
A/common crane/Yunnan-Huize/27/2021(H5N8)
A/common crane/Yunnan-Huize/11/2021(H5N8)
A/common crane/Yunnan-Huize/22/2021(H5N8)
A/Cygnus columbianus/Hubei/116/2020(H5N8)
A/goose/Omsk/01171/2020(H5N8)
A/mute swan/North Ossetia-Alania/325-01/2020(H5N8)
A/chicken/Chelyabinsk/404/2020(H5N8)
A/swan/Kazakhstan/9-20-B-Talg-39/2020(H5N8)
A/chicken/Saratov/29801/2020(H5N8)
A/chicken/France/20P016448/2020(H5N8)
A/goose/Omsk/0002/2020(H5N8)
A/chicken/Tyumen/302-01/2020(H5N8)
A/chicken/Kosovo/22-59 22VIR3124-20/2022(H5N8)
A/chicken/Kosovo/22-9 22VIR3124-15/2022(H5N8)
A/common quail/Kosovo/304-2-22VIR3124-12/2021(H5N8)
A/chicken/Poland/448/2020(H5N8)
A/peregrine falcon/Ireland/20VIR7872-1/2020(H5N8)
A/chicken/Iraq/1/2020(H5N8)
A/chicken/Egypt/F17230B/2019(H5N8)
A/Turkey/Egypt/AI20285/2019(H5N8)
A/Fujian-Sanyuan/21099/2017(H5N6)
A/chicken/Germany.BW/AI00049/2020(H5N8)
A/steamer duck/Germany-SN/AI00346/2020(H5N8)
A/chicken/Czech Republic/1175-1/2020(H5N8)
A/turkey/Poland/23/2019(H5N8)
A/turkey/Czech Republic/3071/2020(H5N8)
A/Duck/Hungary/18444/2020(H5N8)
A/guinea fowl/Nigeria/OG-GF11T 19VIR8424-7/2019(H5N8)
A/Guinea fowl/South Africa/17080274/2017(H5N8)
A/common teal/Korea/W547/2016(H5N8)
A/chicken/Kostroma/1718/2017(H5N2)
A/Bar-headed Goose/Qinghai/a210/2017(H5N8)
A/Bar-headed Goose/Qinghai/BTY1-B/2016(H5N8)
A/Enviroment/Xuzhou/461/2017(H5N6)
A/chicken/Thanh Hoa/VTC741/2020(H5N6)
A/Whooper swan/Mongolia/25/2020(H5N6)
A/Guangdong/18SF020/2018(H5N6)
A/chicken/Thanh Hoa/13836VTC/2019(H5N6)
A/Guangxi/13486/2017(H5N6)
A/Sichuan/26221/2014(H5N6)
A/duck/Hunan/01.21 YYFQH019-P/2015(H5N6)
A/environment/Zhenjiang/C13/2013(H5N6)
A/gyrfalcon/Washington/41088-6/2014(H5N8)
A/chicken/California/15-004912/2015(H5N8)
A/mallard/Alaska/AH0088535/2016(H5N2)
A/duck/Taiwan/A3400/2015(H5N8)
A/crane/Kagoshima/KU1/2014(H5N8)
subgroup A
subgroup B
2.3.4.4b
2.3.4.4h
2.3.4.4a
2.3.4.4c
0.01

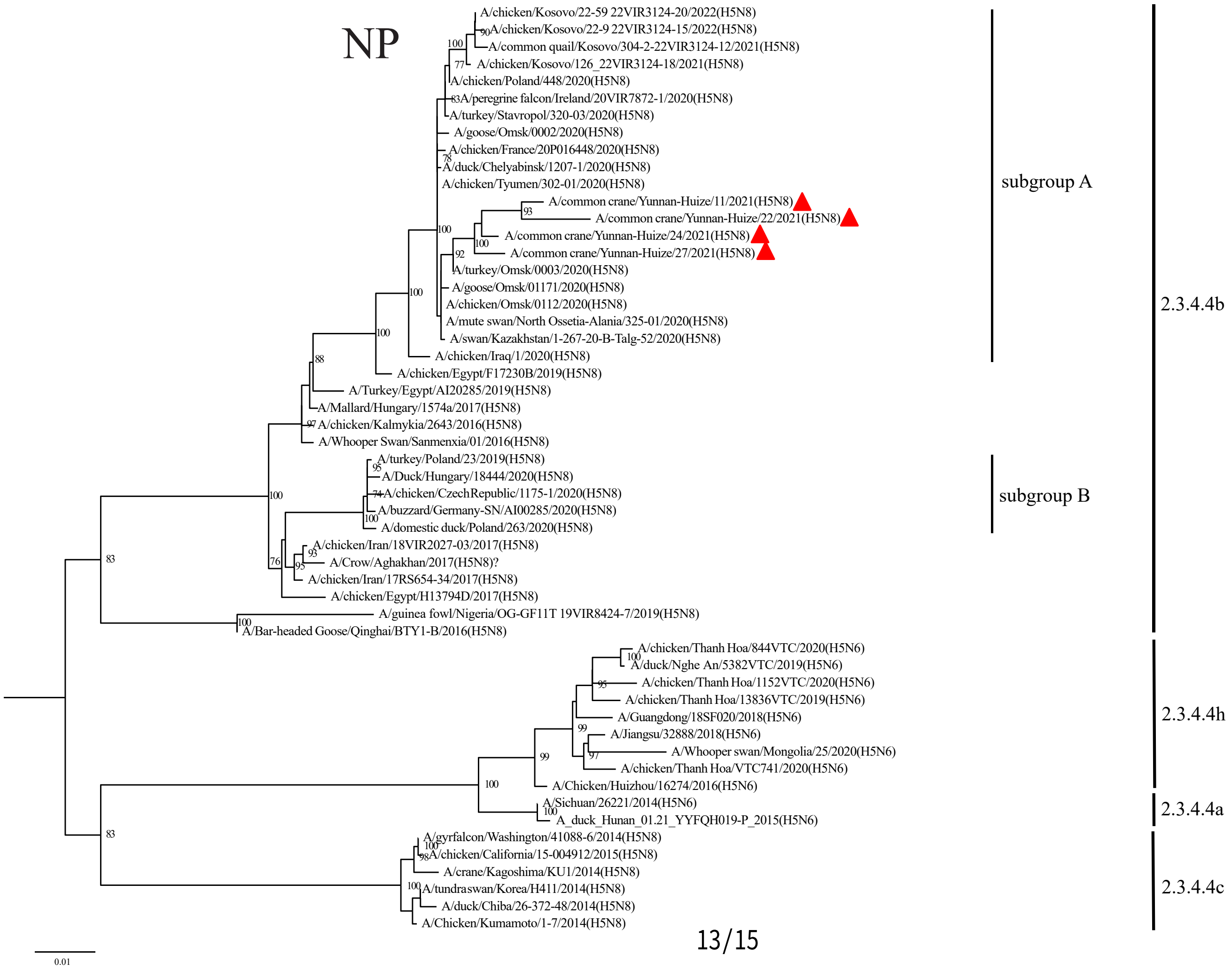
NP
A/chicken/Kosovo/22-59 22VIR3124-20/2022(H5N8)
A/chicken/Kosovo/22-9 22VIR3124-15/2022(H5N8)
A/common quail/Kosovo/304-2-22VIR3124-12/2021(H5N8)
A/chicken/Kosovo/126_22VIR3124-18/2021(H5N8)
A/chicken/Poland/448/2020(H5N8)
A/peregrine falcon/Ireland/20VIR7872-1/2020(H5N8)
A/turkey/Stavropol/320-03/2020(H5N8)
A/goose/Omsk/0002/2020(H5N8)
A/chicken/France/20P016448/2020(H5N8)
A/duck/Chelyabinsk/1207-1/2020(H5N8)
A/chicken/Tyumen/302-01/2020(H5N8)
A/common crane/Yunnan-Huize/11/2021(H5N8)
A/common crane/Yunnan-Huize/22/2021(H5N8)
A/common crane/Yunnan-Huize/24/2021(H5N8)
A/common crane/Yunnan-Huize/27/2021(H5N8)
A/turkey/Omsk/0003/2020(H5N8)
A/goose/Omsk/01171/2020(H5N8)
A/chicken/Omsk/0112/2020(H5N8)
A/mute swan/North Ossetia-Alania/325-01/2020(H5N8)
A/swan/Kazakhstan/1-267-20-B-Talg-52/2020(H5N8)
A/chicken/Iraq/1/2020(H5N8)
A/chicken/Egypt/F17230B/2019(H5N8)
A/Turkey/Egypt/AI20285/2019(H5N8)
A/Mallard/Hungary/1574a/2017(H5N8)
A/chicken/Kalmykia/2643/2016(H5N8)
A/Whooper Swan/Sanmenxia/01/2016(H5N8)
A/turkey/Poland/23/2019(H5N8)
A/Duck/Hungary/18444/2020(H5N8)
A/chicken/CzechRepublic/1175-1/2020(H5N8)
A/buzzard/Germany-SN/AI00285/2020(H5N8)
A/domestic duck/Poland/263/2020(H5N8)
A/chicken/Iran/18VIR2027-03/2017(H5N8)
A/Crow/Aghakhan/2017(H5N8)?
A/chicken/Iran/17RS654-34/2017(H5N8)
A/chicken/Egypt/H13794D/2017(H5N8)
A/guinea fowl/Nigeria/OG-GF11T 19VIR8424-7/2019(H5N8)
A/Bar-headed Goose/Qinghai/BTY1-B/2016(H5N8)
A/chicken/Thanh Hoa/844VTC/2020(H5N6)
A/duck/Nghe An/5382VTC/2019(H5N6)
A/chicken/Thanh Hoa/1152VTC/2020(H5N6)
A/chicken/Thanh Hoa/13836VTC/2019(H5N6)
A/Guangdong/18SF020/2018(H5N6)
A/Jiangsu/32888/2018(H5N6)
A/Whooper swan/Mongolia/25/2020(H5N6)
A/chicken/Thanh Hoa/VTC741/2020(H5N6)
A/Chicken/Huizhou/16274/2016(H5N6)
A/Sichuan/26221/2014(H5N6)
A_duck_Hunan_01.21_YYFQH019-P_2015(H5N6)
A/gyrfalcon/Washington/41088-6/2014(H5N8)
A/chicken/California/15-004912/2015(H5N8)
A/crane/Kagoshima/KU1/2014(H5N8)
A/tundra swan/Korea/H411/2014(H5N8)
A/duck/Chiba/26-372-48/2014(H5N8)
A/Chicken/Kumamoto/1-7/2014(H5N8)
subgroup A
subgroup B
2.3.4.4b
2.3.4.4h
2.3.4.4a
2.3.4.4c
0.01

cont.

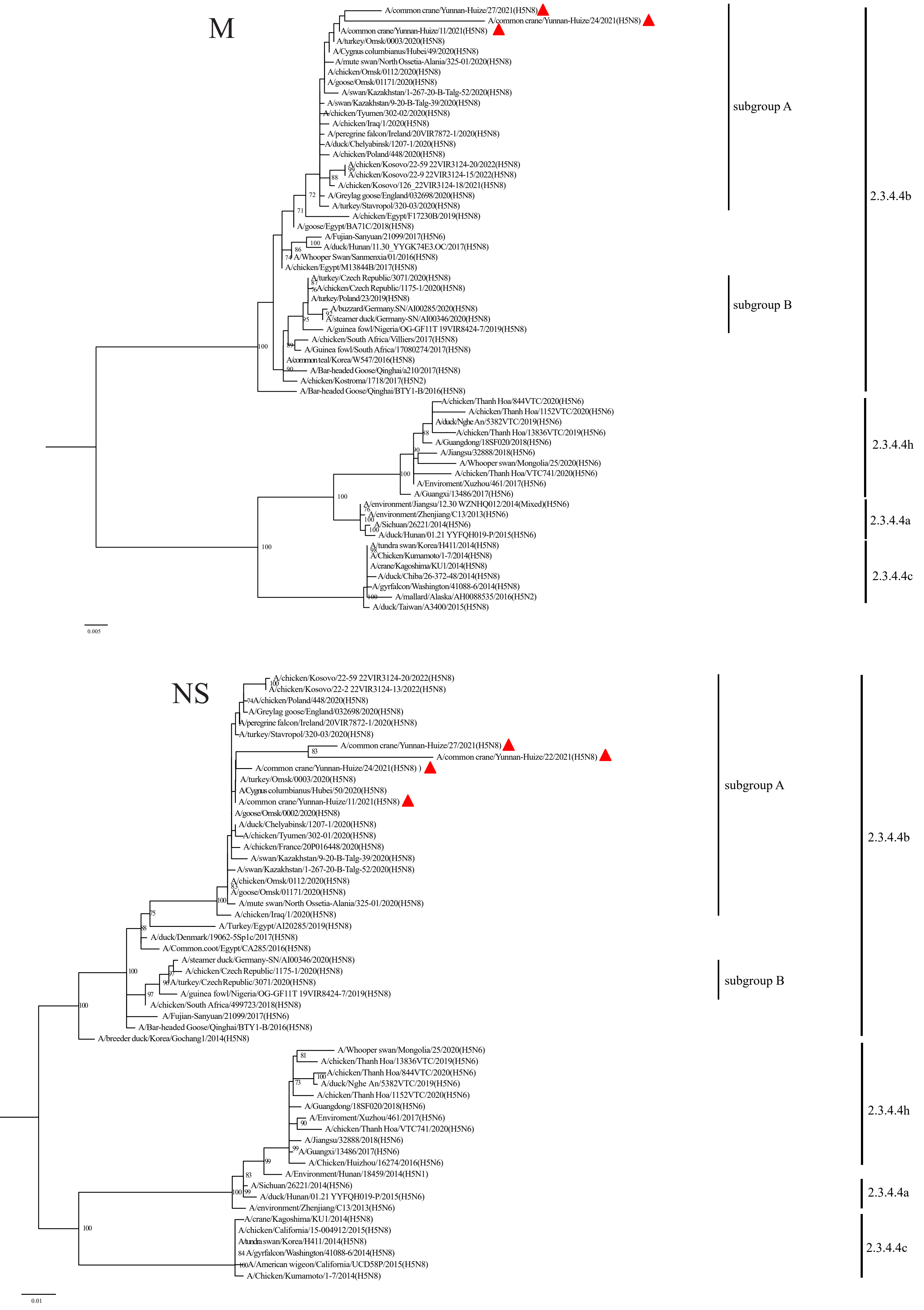

M
A/common crane/Yunnan-Huize/27/2021(H5N8)
A/common crane/Yunnan-Huize/24/2021(H5N8)
A/common crane/Yunnan-Huize/11/2021(H5N8)
A/turkey/Omsk/0003/2020(H5N8)
A/Cygnus columbianus/Hubei/49/2020(H5N8)
A/mute swan/North Ossetia-Alania/325-01/2020(H5N8)
A/chicken/Omsk/0112/2020(H5N8)
A/goose/Omsk/01171/2020(H5N8)
A/swan/Kazakhstan/1-267-20-B-Talg-52/2020(H5N8)
A/swan/Kazakhstan/9-20-B-Talg-39/2020(H5N8)
A/chicken/Tyumen/302-02/2020(H5N8)
A/chicken/Iraq/1/2020(H5N8)
A/peregrine falcon/Ireland/20VIR7872-1/2020(H5N8)
A/duck/Chelyabinsk/1207-1/2020(H5N8)
A/chicken/Poland/448/2020(H5N8)
A/chicken/Kosovo/22-59 22VIR3124-20/2022(H5N8)
A/chicken/Kosovo/22-9 22VIR3124-15/2022(H5N8)
A/chicken/Kosovo/126_22VIR3124-18/2021(H5N8)
A/Greylag goose/England/032698/2020(H5N8)
A/turkey/Stavropol/320-03/2020(H5N8)
A/chicken/Egypt/F17230B/2019(H5N8)
A/goose/Egypt/BA71C/2018(H5N8)
A/Fujian-Sanyuan/21099/2017(H5N6)
A/duck/Hunan/11.30_YYGK74E3.OC/2017(H5N8)
A/Whooper Swan/Sanmenxia/01/2016(H5N8)
A/chicken/Egypt/M13844B/2017(H5N8)
A/turkey/Czech Republic/3071/2020(H5N8)
A/chicken/Czech Republic/1175-1/2020(H5N8)
A/turkey/Poland/23/2019(H5N8)
A/buzzard/Germany.SN/AI00285/2020(H5N8)
A/steamer duck/Germany-SN/AI00346/2020(H5N8)
A/guinea fowl/Nigeria/OG-GF11T 19VIR8424-7/2019(H5N8)
A/chicken/South Africa/Villiers/2017(H5N8)
A/Guinea fowl/South Africa/17080274/2017(H5N8)
A/common teal/Korea/W547/2016(H5N8)
A/Bar-headed Goose/Qinghai/a210/2017(H5N8)
A/chicken/Kostroma/1718/2017(H5N2)
A/Bar-headed Goose/Qinghai/BTY1-B/2016(H5N8)
A/chicken/Thanh Hoa/844VTC/2020(H5N6)
A/chicken/Thanh Hoa/1152VTC/2020(H5N6)
A/duck/Nghe An/5382VTC/2019(H5N6)
A/chicken/Thanh Hoa/13836VTC/2019(H5N6)
A/Guangdong/18SF020/2018(H5N6)
A/Jiangsu/32888/2018(H5N6)
A/Whooper swan/Mongolia/25/2020(H5N6)
A/chicken/Thanh Hoa/VTC741/2020(H5N6)
A/Enviroment/Xuzhou/461/2017(H5N6)
A/Guangxi/13486/2017(H5N6)
A/environment/Jiangsu/12.30 WZNHQ012/2014(Mixed)(H5N6)
A/environment/Zhenjiang/C13/2013(H5N6)
A/Sichuan/26221/2014(H5N6)
A/duck/Hunan/01.21 YYFQH019-P/2015(H5N6)
A/tundra swan/Korea/H411/2014(H5N8)
A/Chicken/Kumamoto/1-7/2014(H5N8)
A/crane/Kagoshima/KU1/2014(H5N8)
A/duck/Chiba/26-372-48/2014(H5N8)
A/gyrfalcon/Washington/41088-6/2014(H5N8)
A/mallard/Alaska/AH0088535/2016(H5N2)
A/duck/Taiwan/A3400/2015(H5N8)
subgroup A
subgroup B
2.3.4.4b
2.3.4.4h
2.3.4.4a
2.3.4.4c
0.005
NS
A/chicken/Kosovo/22-59 22VIR3124-20/2022(H5N8)
A/chicken/Kosovo/22-2 22VIR3124-13/2022(H5N8)
A/chicken/Poland/448/2020(H5N8)
A/Greylag goose/England/032698/2020(H5N8)
A/peregrine falcon/Ireland/20VIR7872-1/2020(H5N8)
A/turkey/Stavropol/320-03/2020(H5N8)
A/common crane/Yunnan-Huize/27/2021(H5N8)
A/common crane/Yunnan-Huize/22/2021(H5N8)
A/common crane/Yunnan-Huize/24/2021(H5N8))
A/turkey/Omsk/0003/2020(H5N8)
A/Cygnus columbianus/Hubei/50/2020(H5N8)
A/common crane/Yunnan-Huize/11/2021(H5N8)
A/goose/Omsk/0002/2020(H5N8)
A/duck/Chelyabinsk/1207-1/2020(H5N8)
A/chicken/Tyumen/302-01/2020(H5N8)
A/chicken/France/20P016448/2020(H5N8)
A/swan/Kazakhstan/9-20-B-Talg-39/2020(H5N8)
A/swan/Kazakhstan/1-267-20-B-Talg-52/2020(H5N8)
A/chicken/Omsk/0112/2020(H5N8)
A/goose/Omsk/01171/2020(H5N8)
A/mute swan/North Ossetia-Alania/325-01/2020(H5N8)
A/chicken/Iraq/1/2020(H5N8)
A/Turkey/Egypt/AI20285/2019(H5N8)
A/duck/Denmark/19062-5Sp1c/2017(H5N8)
A/Common.coot/Egypt/CA285/2016(H5N8)
A/steamer duck/Germany-SN/AI00346/2020(H5N8)
A/chicken/Czech Republic/1175-1/2020(H5N8)
A/turkey/Czech Republic/3071/2020(H5N8)
A/guinea fowl/Nigeria/OG-GF11T 19VIR8424-7/2019(H5N8)
A/chicken/South Africa/499723/2018(H5N8)
A/Fujian-Sanyuan/21099/2017(H5N6)
A/Bar-headed Goose/Qinghai/BTY1-B/2016(H5N8)
A/breeder duck/Korea/Gochang1/2014(H5N8)
A/Whooper swan/Mongolia/25/2020(H5N6)
A/chicken/Thanh Hoa/13836VTC/2019(H5N6)
A/chicken/Thanh Hoa/844VTC/2020(H5N6)
A/duck/Nghe An/5382VTC/2019(H5N6)
A/chicken/Thanh Hoa/1152VTC/2020(H5N6)
A/Guangdong/18SF020/2018(H5N6)
A/Enviroment/Xuzhou/461/2017(H5N6)
A/chicken/Thanh Hoa/VTC741/2020(H5N6)
A/Jiangsu/32888/2018(H5N6)
A/Guangxi/13486/2017(H5N6)
A/Chicken/Huizhou/16274/2016(H5N6)
A/Environment/Hunan/18459/2014(H5N1)
A/Sichuan/26221/2014(H5N6)
A/duck/Hunan/01.21 YYFQH019-P/2015(H5N6)
A/environment/Zhenjiang/C13/2013(H5N6)
A/crane/Kagoshima/KU1/2014(H5N8)
A/chicken/California/15-004912/2015(H5N8)
A/tundra swan/Korea/H411/2014(H5N8)
A/gyrfalcon/Washington/41088-6/2014(H5N8)
A/American wigeon/California/UCD58P/2015(H5N8)
A/Chicken/Kumamoto/1-7/2014(H5N8)
subgroup A
subgroup B
2.3.4.4b
2.3.4.4h
2.3.4.4a
2.3.4.4c
0.01

cont.

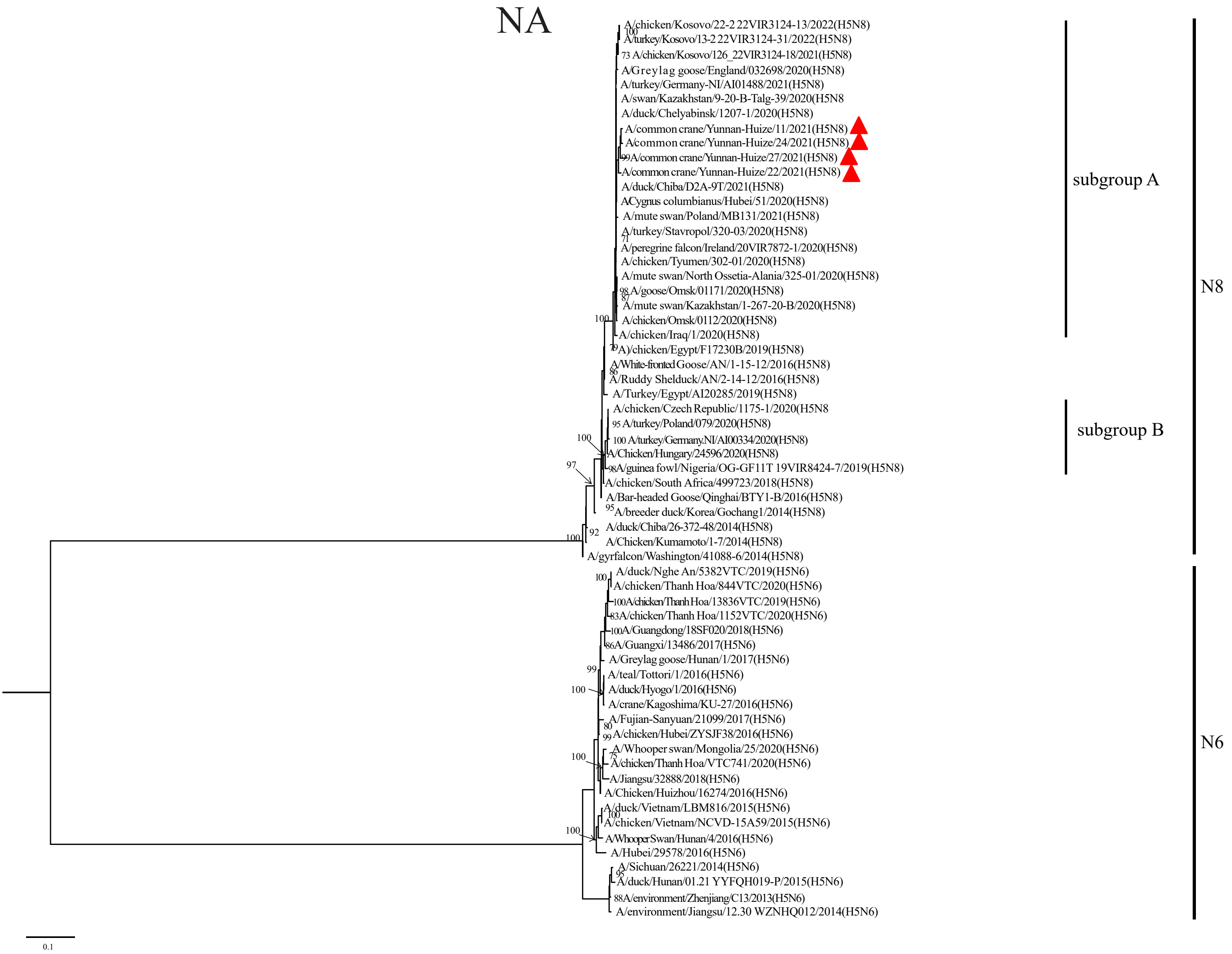

Supplementary Figure S1. Maximum likelihood (ML) phylogenetic trees of PB2 (polymerase basic protein 2), PB1 (polymerase basic protein 1); PA (polymerase acidic protein), NP (nucleoprotein), MP (matrix protein), NS (nonstructural protein) and NA (neuraminidase) gene segments of the H5N8 AIVs. Strains of H5N8 from common cranes in this study were labelled with red triangles. All the seven genes are clustered in clade 2.3.4.4b of H5Nx HPAIVs, and derived from A/Turkey/Egypt/AI20285/2019. Branch supports were assessed with 1 000 bootstrap replicates and only values higher than 70% are shown at the branch nodes.
